# Therapeutic Potential of *Salvia rosmarinus*: Seasonal and Geographical Variation in Phytochemical Composition, Bioactivity, and Synergistic Effects of Rosmarinic Acid with 5-FU

**DOI:** 10.3390/plants15010001

**Published:** 2025-12-19

**Authors:** Mariana Oalđe Pavlović, Milena Milutinović, Ana Alimpić Aradski, Uroš Gašić, Danijela Mišić, Petar D. Marin, Sonja Duletić-Laušević

**Affiliations:** 1 University of Belgrade, Faculty of Biology, Institute of Botany and Botanical Garden “Jevremovac”, Studentski trg 16, 11000 Belgrade, Serbia; alimpic.ana@bio.bg.ac.rs (A.A.A.); pdmarin@bio.bg.ac.rs (P.D.M.); 2 University of Kragujevac, Faculty of Science, Department of Biology and Ecology, Radoja Domanovića 12, 34000 Kragujevac, Serbia; milenagen@gmail.com; 3 University of Belgrade, Institute for Biological Research “Siniša Stanković”, National Institute of the Republic of Serbia, Bulevar Despota Stefana 142, 11000 Belgrade, Serbia; dmisic@ibiss.bg.ac.rs

**Keywords:** *Salvia rosmarinus*, chemical characterization, biological activities, synergistic effects, rosmarinic acid

## Abstract

*Salvia rosmarinus* Spenn. (rosemary) is a medicinal and aromatic plant of notable pharmacological value. This study evaluated the therapeutic properties of rosemary leaves collected from two Serbian continental (L1, L2) and one Montenegrin Mediterranean (L3) locations, harvested in November (N), March (M), and July (J). Extracts prepared with 70% methanol, 70% ethanol, and water were analyzed for chemical composition and biological activity. L3 extracts exhibited the highest polyphenolic content, with L3M methanolic extract showing the greatest total phenolic (134.60 mg GAE/g) and phenolic acid levels (211.96 mg CAE/g), and L3M ethanolic extract the highest flavonoid content (25.54 mg QE/g). LC/MS analysis identified 28 previously unreported compounds in *Rosmarinus* sp. extracts, revealing hydroxycinnamic acid derivatives and flavonoid *O*-glycosides as the main constituents in *S. rosmarinus*. The alcoholic extracts were rich in 1,8-cineole, camphor, borneol, terpinen-4-ol, and verbenone. L3 extracts demonstrated the strongest antioxidant and enzyme-inhibitory activities, often surpassing positive controls. L3J showed pronounced cytotoxicity against HCT-116 colorectal cancer cells (IC_50_ = 13.08 µg/mL after 24 h incubation), while showing non-cytotoxic effects on normal human keratinocytes (IC_50_ > 500 µg/mL). Finally, rosmarinic acid alone synergistically enhanced the cytotoxic effect of 5-fluorouracil (combination index < 0.8). This comprehensive study highlights the influence of geography, season, and solvent on phytochemical profile and bioactivity of rosemary extracts, emphasizing the therapeutic potential of distinct rosemary populations.

## 1. Introduction

Approximately 70,000 species are being used to treat various diseases, yet only around 15% of the total plant species worldwide have undergone systematic investigation for their medicinal potential. Despite this relatively low exploration rate, it is noteworthy that 25% of the conventional medicines employed in modern healthcare have originated from plants [1,2]. This observation implies ample opportunity for additional research on natural sources, indicating that further investigations into the medicinal properties of plants could yield essential contributions to science [3].

The Lamiaceae family is an invaluable source of medicinal and aromatic plants, encompassing species with both historical and modern importance. *Salvia rosmarinus* Spenn. (formerly known as *Rosmarinus officinalis* L.), rosemary, a perennial herb native to the Mediterranean region, plays a significant role in both traditional and contemporary medicinal practices, serving diverse purposes acting as antibacterial, antiepileptic, antirheumatic, antispasmodic, carminative, diuretic, expectorant, renal colic, and wound-healing agent [4,5]. Moreover, this widely used culinary herb not only enhances dishes with its distinct flavor and aroma, but it is also used as a food preservative; therefore, exploring and understanding the biological activities of rosemary is crucial [6].

The biological potential of rosemary is linked with its high content of phenolic compounds, which primarily fall into several classes: phenolic acids (including caffeic and rosmarinic acids), flavonoids (such as cirsimaritin, genkwanin, homoplantaginin, and scutellarein), phenolic diterpenes (such as carnosic acid, carnosol, rosmadial, and rosmanol), and triterpenes (like oleanolic and ursolic acids) [7]. These compounds play a crucial role in displaying rosemary’s antioxidant activity. Furthermore, rosemary is known for its anti-inflammatory effects, reducing the risk of chronic inflammation-related diseases [4,8]. Oxidative stress and inflammatory diseases are interlinked through a complex network of molecular pathways. Free radicals such as reactive oxygen species (ROS) can directly activate inflammatory signaling pathways and immune cells, leading to increased production of pro-inflammatory cytokines and sustained tissue damage. This interaction creates a feedback loop that perpetuates both oxidative stress and inflammation, contributing to the development and progression of various chronic inflammatory diseases, including diabetes, neurodegenerative disorders, cardiovascular diseases, and cancer [9,10]. Rosemary’s phytoconstituents, like carnosol and carnosic acid, are known to reduce the risk of diseases associated with chronic inflammation. They inhibit pro-inflammatory enzymes (COX-2, LOX) and cytokines (TNF-α, IL-6), and modulate key signaling pathways (e.g., NF-κB), thereby mitigating inflammatory responses [8,11,12]. Moreover, studies have highlighted that rosemary extracts, rich in phenolic compounds, can also improve insulin sensitivity, enhance glucose uptake, and protect neurons from oxidative stress, making them promising for managing diabetes and reducing Alzheimer’s disease risk [4,8,12]. The antitumor activity of rosemary has been associated with multiple mechanisms, including antioxidant and antiangiogenic effects, epigenetic modulation, and alteration of specific metabolic pathways, downregulation of oncogenes, and upregulation of tumor suppressor genes [13].

Excess ROS also result in damage to DNA, proteins, and lipids, leading to genetic mutations, disruption of cellular signaling, and promotion of tumor growth. Additionally, due to their rapid metabolism, as well as high proliferation rate, cancer cells increase their production of ROS [9]. On the other hand, plant extracts are known to neutralize ROS, reduce oxidative stress, and interfere with the development and progression of cancer. Hence, rosemary extracts and isolated compounds have been extensively studied for their ability to induce apoptosis, inhibit angiogenesis, and enhance the efficacy of conventional chemotherapy [7,14,15]. Studies suggest that the combined use of rosemary extracts and/or their phytoconstituents with chemotherapeutic drugs displays synergistic effects, enhancing drug efficacy while reducing resistance and minimizing side effects [16]. This integrative approach leverages the bioactive compounds in plants to improve treatment outcomes and patients’ quality of life.

Considering the existing reports regarding rosemary’s potent bioactive properties, this study was designed to systematically analyze, compare, and comprehensively evaluate the chemical composition and biological activities of methanolic, ethanolic, and aqueous extracts of this species from two continental climates in Serbia (Belgrade and Bogatić), and one Mediterranean climate in Montenegro (Lastva Grbaljska), harvested in three different seasons (November, March, and July). The study aimed to assess the chemical profile, as well as the antioxidant, hypoglycemic, and antineurodegenerative activities of these extracts to identify variations due to extraction solvents, seasonal changes, and geographical differences. Additionally, the extracts harvested in Lastva Grbaljska were selected for the evaluation of their cytotoxic activities on a colorectal cell line, HCT-116. The final step of this study was to determine the potential interactions between rosmarinic acid and 5-fluorouracil (5-FU), a commonly used chemotherapeutic for colorectal carcinoma, providing a holistic understanding of rosemary’s potential as a therapeutic agent.

## 2. Results

### 2.1. Yield of Rosemary Extracts

The highest extraction yield was observed in the aqueous extracts, followed closely by the ethanolic extracts, while the methanolic extracts showed the lowest yield (Table 1). Among all the extracts, the L3M aqueous extract had the highest yield at 18.70%. In contrast, the lowest yield was found in the L1N and L2J ethanolic extracts (8.77 and 8.80%, respectively).

### 2.2. Chemical Content and LC/MS Analysis of Rosemary Extracts

Since the results demonstrated a concentration-dependent trend, the phytochemical content is presented only for the extracts tested at 500 μg/mL. Among all extracts, the highest levels of the quantified secondary metabolites were found in the methanolic extracts from Lastva Grbaljska, Montenegro, followed by the ethanolic extracts from Belgrade, Serbia. In contrast, the aqueous extracts from Bogatić, Serbia, consistently showed the lowest content across all phenolic groups (Table 2). Specifically, the L3M methanolic extract exhibited the highest TPC (134.60 mg GAE/g), while the L2J aqueous extract had the lowest TPC (30.38 mg GAE/g). Statistical analysis indicated significant differences in TPC depending on both the locality and the solvent used. Additionally, there was a significant difference in TPC between the plant material harvested in March and July, with the March harvest showing higher TPC values.

The L3M methanolic extract also had the highest PAC (211.96 mg CAE/g), whereas the L2J aqueous extract had the lowest PAC (21.22 mg CAE/g). Both the locality and the solvent type influenced the PAC levels in the extracts. Notably, significant differences were observed between plant material harvested in March and July, with the March harvest displaying higher PAC levels (Table 2).

The highest TFC was found in the L3M ethanolic extract (25.54 mg QE/g) and L1M methanolic extract (25.51 mg QE/g), while the lowest TFC was again recorded in the L2J aqueous extract (5.44 mg QE/g). The statistical analysis revealed that the TFC was significantly affected by the locality. Moreover, the March harvest displayed higher TFC levels than the July harvest. Methanolic extracts were significantly richer in these secondary metabolites than the aqueous extracts (Table 2).

The highest FC was observed in the L3N ethanolic extract (5.73 mg QE/g), while no FC was detected in the L2J aqueous extract. Both the locality and the solvent type influenced the FC levels. Specifically, L1 and L3 extracts had similar FC levels, while L2 extracts had significantly lower FC. Finally, the methanolic and ethanolic extracts had comparable FC levels, while the aqueous extracts had significantly lower FC (Table 2).

GC/MS profiling of volatile terpenoids in rosemary extracts revealed a total of 28 compounds belonging to the following groups: monoterpenes (11 compounds), sesquiterpenes (7 compounds), diterpenes (8 compounds), and triterpenes (2 compounds) (Table S1). Monoterpene hydrocarbons are represented by 4 compounds (α-phellandrene, 1,3,8-p-menthatriene, β-terpinen, and β-phellandrene), while 7 oxygenated monoterpenoides (1,8-cineole, camphor, borneol, terpinen-4-ol, a-terpineol, verbenone, and thymol) were present in the analyzed rosemary samples. Of the analyzed solvents, ethanol was the most efficient for the extraction of monoterpenes, especially of monoterpene hydrocarbons. Methanol was also efficient in extracting oxygenated monoterpenoides.

Ethanol-extracted samples were especially rich in sesquiterpene hydrocarbons (α-amorphene, δ-cadinene, α-calacorene, and 1,4-dimethyl-7-(1-methylethyl)-azulene) and oxygenated sesquiterpenoids (caryophyllene oxide, α-santalol, caryophyllenyl alcohol). Methanol and water were much less efficient in extracting rosemary sesquiterpenes. As for simple diterpenes (abietadiene, abitatriene) and oxygenated diterpenoides (epi-13-manool, ferruginol, carnosol, (−)-20-deoxocarnosol, pisiferal, pisiferol) identified in rosemary extracts, ethanol was the most efficient extraction solvent. Triterpenes (β-amyrin and α-amyrin) identified in rosemary samples were only found in ethanolic and aqueous extracts. Methanol was less efficient in extracting triterpenes (Table S1).

Using the hybrid LC/MS technique, a total of 92 different compounds were identified in the 27 tested rosemary extracts (Table S2). The given compounds are divided into groups according to their chemical diversity (Table S3): 8 derivatives of hydroxybenzoic acids, 30 derivatives of hydroxycinnamic acids, 31 flavonoid glycosides, 9 flavonoid aglycones, 5 terpenoids, and 9 compounds classified as other metabolites. Tentative identification of the compounds was carried out by the study of their exact masses, which were obtained from full scan MS spectra, and SciFinder search by molecular formula was used as a reference database [17]. Based on the high-resolution MS^2^ fragmentation, the chemical structures of the identified compounds were proposed. Table S3 also includes references [18,19,20,21,22,23,24,25,26,27,28,29,30,31,32,33,34,35,36,37,38,39,40] documenting previous isolation/identification of each compound in various *Rosmarinus* species, and highlights 28 compounds reported here for the first time in *Rosmarinus* sp. extracts.

### 2.3. Antioxidant Activity of Rosemary Extracts

The antioxidant activity of rosemary extracts was tested using DPPH, FRAP, TRP, and β-carotene bleaching assays, and the results are shown at a tested concentration of 250 μg/mL (Table 3). The March harvest of the rosemary plants from Lastva Grbaljska generally showed the highest antioxidant activity, except in the β-carotene bleaching assay, where the November harvest exhibited the strongest effect. However, no statistically significant differences were observed in the antioxidant activity related to the harvesting season. Overall, alcoholic extracts, particularly methanolic ones, demonstrated the most potent antioxidant properties, except in the β-carotene bleaching assay, where the ethanolic extracts were the most effective (Table 3).

In the DPPH assay, the L3N methanolic extract exhibited the highest radical-scavenging activity (89.99%), which was statistically similar to the positive control, ascorbic acid (89.70%). Several alcoholic extracts from various localities (L1N, L1M, L2M, L3N, L3M, L3J methanolic and ethanolic, and L2N and L2J methanolic extracts), along with two aqueous extracts (L3N and L3J), showed higher activity than the positive controls BHA and BHT (55.43 and 52.01%, respectively). Additionally, aqueous extracts from March harvest (L1M, L2M, and L3M) were more active than BHT. Rosemary harvested in Lastva Grbaljska exhibited significantly higher DPPH-scavenging activity than the material from Belgrade and Bogatić, while the methanolic and ethanolic extracts outperformed aqueous extracts. The lowest DPPH activity was observed for L2J aqueous extract (25.80%) (Table 3).

The highest ferric-reducing capacity was found in the L3N methanolic extract (873.58 μg FeSO_4_ × 7H_2_O/g), with L3M and L3J methanolic extracts also showing significantly higher activity than the positive control BHT (559 μg FeSO_4_ × 7H_2_O/g). The lowest activity was noted for the L2J aqueous extract (207.35 μg FeSO_4_ × 7H_2_O/g). Rosemary from Lastva Grbaljska had significantly higher ferric-reducing potential than those from Belgrade and Bogatić. The methanolic extracts exhibited significantly higher antioxidant activity than the ethanolic and aqueous extracts. However, rosemary extracts had significantly lower activity than the positive controls, BHA and ascorbic acid (Table 3).

The highest TRP was found for L3M methanolic extract (1182.39 μg AAE/g), while the lowest TRP was observed for L2N and L2J aqueous extracts (267.85 μg AAE/g). The rosemary from Lastva Grbaljska displayed significantly higher TRP than the material from Belgrade and Bogatić, with the methanolic extracts showing the highest reducing power among the solvents tested (Table 3).

The L1J ethanolic extract was the most effective in protecting β-carotene from bleaching (74.33%), significantly outperforming the positive controls BHA, BHT, and ascorbic acid (64.47, 59.12, and 6.92%, respectively). Although the L1J aqueous extract exhibited the lowest activity (39.27%), it still outperformed the ascorbic acid at same concentration (6.92%). Generally, all rosemary extracts showed significantly greater inhibition of β-carotene bleaching compared to ascorbic acid. Although the rosemary from Lastva Grbaljska was more active than the material from Belgrade and Bogatić, the difference was not statistically significant. Notably, the ethanolic extracts provided the best protection against β-carotene bleaching, followed by the methanolic and aqueous extracts (Table 3).

### 2.4. Enzyme Inhibitory Activity of Rosemary Extracts

The results of the enzyme inhibitory activity of rosemary extracts are shown at a tested concentration of 1 mg/mL for the α-glucosidase inhibition assay, and at 250 μg/mL for the AChE and TYR assays. Overall, the rosemary from Lastva Grbaljska demonstrated the highest enzyme inhibitory activity. No statistically significant differences were observed between the results from the plant material harvested in November, March, and July (Table 4).

In the α-glucosidase assay, the highest activity was observed for the L3N ethanolic and L3M and L3J aqueous extracts (98.64, 98.17%, and 98.10%, respectively). These results, along with those of several other extracts, were significantly higher than the positive control, acarbose (58.05%). Rosemary from Lastva Grbaljska exhibited significantly stronger inhibition of α-glucosidase activity than the material from Belgrade and Bogatić. Aqueous extracts generally showed significantly lower α-glucosidase activity than the alcoholic extracts, and the L2M aqueous extract exhibited no measurable activity at 1 mg/mL (Table 4).

The L2J and L1J aqueous extracts displayed the highest AChE inhibitory activity (86.30% and 86.24%, respectively), significantly outperforming the positive control, galantamine (63.21%). Among the different harvesting locations, rosemary from Lastva Grbaljska was the most potent AChE inhibitor, followed by the material from Bogatić, while the material from Belgrade was the least effective. The aqueous extracts exerted significantly higher anti-AChE activity than the alcoholic extracts, with the methanolic extract being the least active. The L1N methanolic extract displayed the lowest AChE inhibitory activity (22.58%) (Table 4).

For TYR inhibition, the L2J methanolic extract exhibited the highest activity (60.63%), significantly outperforming the positive control, kojic acid (45.59%). Statistical analysis indicated that the rosemary from Lastva Grbaljska had a statistically higher potential to inhibit TYR activity than the plant material from Belgrade and Bogatić. However, there were no significant differences in TYR inhibition between the different harvest seasons or solvents used. The L2J aqueous extract was the least effective, inhibiting only 37.04% of TYR activity (Table 4).

### 2.5. The IBR Analysis of Antioxidant and Enzyme-Inhibitory Activities of Rosemary Extracts

**Figure 1:**
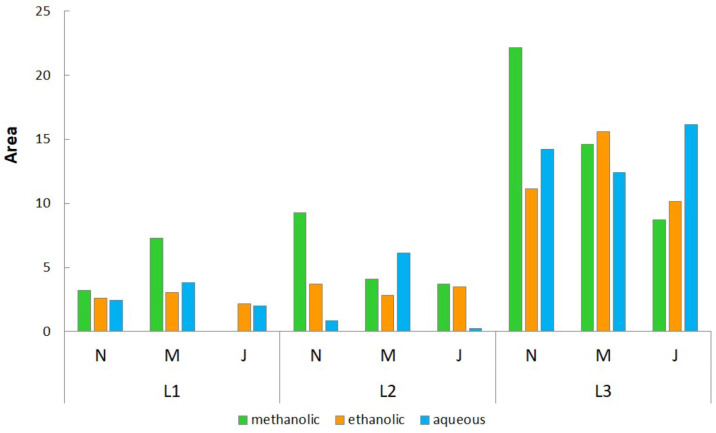
The biological potential of the rosemary extracts obtained by the IBR analysis of standardized results from the four antioxidant assays and three enzyme-inhibitory assays.

The IBR analysis of standardized results from the four antioxidant assays and three enzyme-inhibitory assays is presented in Figure 1 and Figure S1. Overall, the methanolic extract of rosemary harvested in November from Lastva Grbaljska displayed the highest bioactivity, while the methanolic extract prepared from the plant material harvested in Belgrade in the month of July had the lowest activity. Among the different localities, the rosemary samples from Lastva Grbaljska showed the highest activity, followed by those from Bogatić and Belgrade. Regarding the harvest time, the November harvest was the most active for Bogatić and Lastva Grbaljska samples, while the March harvest was the most active for Belgrade samples. The July harvest consistently showed the lowest activity. Methanolic extracts demonstrated the strongest bioactivity, followed by aqueous extracts (except for Bogatić samples), with ethanolic extracts showing the lowest activity (except for Bogatić samples).

Due to their high biological potential demonstrated in the experiments above, rosemary extracts of the plant material harvested in Lastva Grbaljska were further evaluated for their anticancer activity.

### 2.6. Anticancer Properties of Rosemary Extracts

The extracts with the highest concentration of phenolics and bioactivity, prepared from the plant material harvested in the Mediterranean climate of Lastva Grbaljska (L3N, L3M, L3J), were further evaluated for their anticancer properties. Their cytotoxicity was tested on HCT-116 colorectal cancer cells, in parallel with their effects on normal HaCaT keratinocytes. The extracts inhibited the growth of HCT-116 cells, with methanolic extracts showing the highest anticancer potential, followed by ethanolic extracts (Figure S2). IC_50_ values presented in Table 5, and calculated from cell viability curves, indicate remarkable cytotoxicity, with many values below 30 µg/mL (a benchmark proposed by the American National Cancer Institute (NCI) to define cytotoxicity in crude extracts) [41]. The results indicate that water was notably less effective in isolating bioactive substances with anticancer potential from rosemary compared to when alcoholic solvents were used. Among the extracts from different seasons, the alcoholic extracts obtained from the July harvest exhibited the most significant cytotoxicity after 24 h of incubation (the results with the lowest IC_50_ values), while after 72 h of incubation, the most active were the alcoholic extracts obtained from the November harvest. Meanwhile, rosmarinic acid, one of the major constituents in the extracts, was also tested individually, and has shown to induce moderate cytotoxic activity on the cancer cells (Table 5).

All tested extracts, as well as rosmarinic acid, demonstrated selectivity, showing non-cytotoxic effects on normal human keratinocytes. Any observed reduction in cell viability occurred only at the highest applied concentration, with all calculated IC_50_ values exceeding 500 µg/mL for the extracts, and 250 µg/mL for rosmarinic acid, indicating non-cytotoxic activity on normal human keratinocytes (Table 5).

### 2.7. The IBR Analysis of Antioxidant, Enzyme-Inhibitory, and Cytotoxic Activities of Lastva Grbaljska Extracts

**Figure 2:**
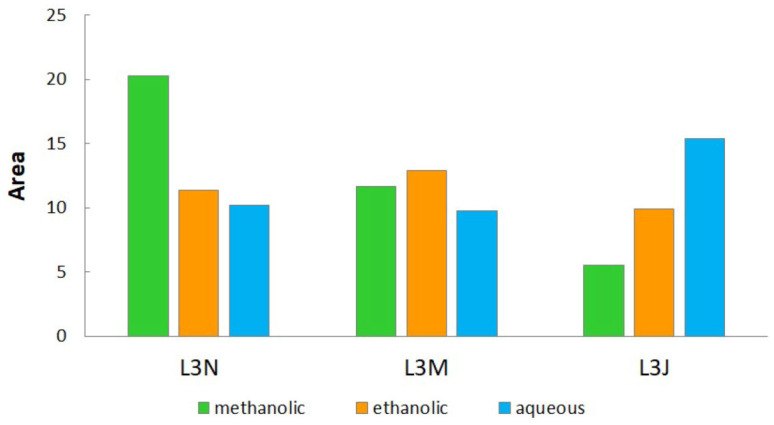
The biological potential of the rosemary extracts of the plant material harvested in Lastva Grbaljska obtained by the IBR analysis of standardized results from the four antioxidant assays and three enzyme-inhibitory assays, and a cytotoxicity assay.

The IBR analysis, shown in Figure 2 and Figure S3, evaluated the effectiveness of rosemary extracts from Lastva Grbaljska in terms of antioxidant, enzyme-inhibitory, and cytotoxic activities across eight assays. The results revealed that the methanolic extract from the November harvest exhibited the highest overall bioactivity, while the methanolic extract from the July harvest was the least active one. This indicates that the November harvest had the greatest biological potential, followed by the March and July harvests. Among the solvents used, methanolic extracts were the most active overall, followed by aqueous extracts, with ethanolic extracts showing the lowest activity.

### 2.8. Synergistic Interactions of Rosmarinic Acid and 5-FU

Our results indicated that rosmarinic acid was among the most abundant phytochemicals identified in the investigated extracts (Table S2), which led to its selection for further evaluation of potential synergistic interactions with 5-FU. To investigate the potential synergistic interactions of rosmarinic acid and 5-FU, these compounds were applied in a combined treatment, where 1 µg/mL of 5-FU was added to each concentration of rosmarinic acid. The IC_50_ values indicate that the combined treatment significantly enhanced the cytotoxicity (Table 6) compared to rosmarinic acid alone (Table 5). Moreover, the CI values of less than 0.8 indicated strong synergistic interactions across all combined concentrations and time points (24 and 72 h). No additive effects or antagonistic interactions were observed.

## 3. Discussion

The present study is distinguished by its sampling design, encompassing rosemary plant material collected from three geographically distinct locations, namely, Belgrade and Bogatić (Serbia), as well as Lastva Grbaljska (Montenegro), and across three different seasons (November, March, and July). November, March, and July were selected as harvesting seasons because they represent three distinct seasonal and phenological phases: late autumn, early spring, and midsummer, characterized by contrasting temperature, light intensity, and precipitation. These environmental differences are known to influence the biosynthesis of polyphenols, terpenoids, and other secondary metabolites in *Salvia rosmarinus*. Additionally, these months correspond to common regional harvest periods, making them ecologically and agriculturally relevant. Sampling during these intervals enabled us to capture realistic seasonal variation in the phytochemical composition and biological activity of rosemary. The approach we opted for in this research provides a unique opportunity to evaluate the combined influence of spatial and temporal factors on the phytochemical composition and bioactivity of rosemary. However, the interpretation of the obtained data is inherently complex, given the large number of samples and the multifactorial nature of the investigated variables. Nevertheless, this extensive and systematic design significantly enhances the scientific relevance and novelty of the study, offering a more integrated understanding of the natural variability and bioactive potential of this plant.

Rosemary is widely recognized for its rich phytochemical composition, particularly its high levels of phenolic and terpenoid compounds, which underlie its diverse biological activities [4,7,12,16]. In the present study, rosemary extracts demonstrated significant antioxidant, enzyme-inhibitory, and cytotoxic activities across various chemical, biochemical, and cell-based assays, consistent with their phytochemical richness. GC/MS analysis revealed 28 volatile terpenoids, including 1,8-cineole, camphor, borneol, and *β*-pinene, compounds previously reported to contribute to the antimicrobial, antioxidant, anti-inflammatory, and antitumor effects of rosemary [42]. Furthermore, LC/MS profiling identified 92 distinct metabolites encompassing phenolic acids, flavonoids, terpenoids, and other minor constituents, with 28 compounds reported for the first time in *Rosmarinus* sp. extracts (Table S3). Notably, several newly detected metabolites, such as aesculetin, have previously been associated with notable pharmacological properties and health-promoting effects [43].

The results of this study show that the methanolic extracts had the highest content of phytoconstituents overall, followed by the ethanolic and aqueous extracts, which is partly in contrast to the results obtained by Al-Jaafreh [44]. The author found that the ethanolic extracts of rosemary from Jordan have a higher TPC and TFC content than the methanolic extract, while the aqueous extract had the lowest TPC and TFC. In our study, the rosemary harvested in Lastva Grbaljska had the highest content of the tested phytochemicals, followed by the plant material harvested in Belgrade and Bogatić. On the other hand, the plant material harvested in March had the highest TPC, PAC, and TFC, followed by the November and July harvests (Table 2). It is well known that the phytochemical content in plant extracts is influenced by the choice of extraction method, growth locality, and harvesting season, among others [45]. Earlier studies confirmed that solvent choice significantly impacts the polyphenolic content, with methanolic extracts consistently yielding the highest levels of polyphenols, followed by ethanolic and aqueous extracts. Methanol is often preferred as an extraction solvent for maximum polyphenolic extraction, while water might be used when milder extraction of specific phenolic acids is desired [44,46,47]. Furthermore, in our study, higher polyphenolic contents found in the samples harvested in Lastva Grbaljska are not surprising, given the fact that Lastva Grbaljska is situated along the coast of Montenegro and benefits from a Mediterranean climate, which is markedly different from the continental climates of Belgrade and Bogatić in Serbia. Rosemary from these two climates displays notable differences in polyphenolic content due to the climatic and environmental factors influencing their growth, as reported by Li and Zidorn [48]. Higher sunlight exposure of the plant and/or specific mineral-rich soils often results in increased phenolic compounds, as these compounds protect plants from environmental stress. Clear regional variations were observed in the metabolite profiles in samples from Montenegro consistently showing higher levels of several groups of metabolites than samples from Serbia. The most pronounced differences were found with phenolic acids, especially rosmarinic acid, various salvianolic acids and yunnaneic acid derivatives. Diterpenes, including carnosic acid, carnosol and rosmanol also showed significantly higher accumulation in the samples from Montenegro, which can be commented on as the influence of stronger solar radiation and Mediterranean climate conditions. In contrast, flavonoid glycosides (e.g., luteolin, apigenin and quercetin derivatives) showed a more moderate, but still constant, increase in Montenegrin samples, especially during the summer sampling period. Overall, these patterns indicate that rosmarinic acid derivatives and diterpenes are the main contributors to the regional chemical differentiation between Serbian and Montenegrin samples. Moreover, some researchers reported that the polyphenolic content is the highest during late spring and early summer, coinciding with the peak in plants’ metabolic activity and flowering phase. During this period, plants synthesize more phenolic compounds as a defense against increased UV radiation and herbivory, resulting in higher total phenolics [48]. On the other hand, other authors consider the opposite, suggesting that some medicinal plants contain the highest levels of these important phytoconstituents in the winter months and, consequently, that is the season when the harvested plant material exerts the highest biological activity [49]. Although our literature survey showed that there is no universal guideline for the optimal harvest time to maximize yields of specific secondary metabolites, the current study suggests that the highest TPC, TFC, and PAC contained the extracts of the rosemary harvested in March, followed by the November and July harvests, while the July harvest had the highest FC, followed by the November and March harvests, which corroborated previous reports by Luis and Johnson [50] and Kabubii et al. [51].

When assessing the antioxidant activity of the samples, it is important to employ a battery of tests in order to detect various mechanisms of their action [44]. Hence, three groups of tests were applied in our study to assess and compare the antioxidant activity of various rosemary extracts, each detecting different antioxidant mechanisms. The first group includes the DPPH assay, which evaluates the ability of bioactive substances to transfer hydrogen atoms or single electrons [52]. The second group consists of the FRAP and TRP assays, which monitor the reduction of ferric (Fe^3+^) to ferrous ions (Fe^2+^), where antioxidants act as reducing agents [53,54]. The third group includes the β-carotene/linoleic acid assay, also known as the β-carotene bleaching assay. β-carotene, a natural antioxidant pigment, reduces oxidative stress at the molecular and tissue levels. The assay measures how well certain substances protect β-carotene from oxidation and degradation [55]. The results of the current study showed that L3 extracts exhibited the highest overall antioxidant activity, which was not surprising given that rosemary is known to thrive under the stress of Mediterranean climate, as these climate conditions promote the biosynthesis of phenolic compounds involved in protecting the plant against oxidative stress [56]. Specifically, L3N methanolic extract had the highest DPPH-scavenging activity as well as the highest activity in the FRAP assay. The L3M methanolic extract demonstrated the highest TRP, while the L1J ethanolic extract most effectively inhibited β-carotene bleaching. These findings are partially consistent with previous reports and can be explained by the phytochemical profiles of the respective extracts. In particular, we found L3N methanolic extract to be the richest in carnosic acid and borneol, L3M methanolic extract in sagerinic acid and borneol, and L1J ethanolic extract in carnosol and pisiferal (Tables S1 and S2). Carnosic acid and carnosol are well-documented for their potent antioxidant activities [4,56], while sagerinic acid, a potential dimerization product of rosmarinic acid, may also contribute to the observed antioxidant effects [22]. Moreover, Al-Jaafreh [44] reported that the methanolic extract had the highest DPPH-scavenging activity (138.3 mg GAE/g), followed by the aqueous extract, while the ethanolic extract had lower activity (106.64 mg GAE/g). On the other hand, the author reported that in FRAP assay, the ethanolic extract was the most active one, following by the aqueous and methanolic ones (130.5, 125.56, and 126.3 mg AScE/mL), while another ferric reduction assay detected that the aqueous extract had the highest potential, followed by the ethanolic and methanolic ones (144.5, 129.1, and 114.3 mg GAE/g). Actually, it was reported that the number of hydroxyl groups and their polarity, among others, influence the antioxidant activity of phenolic compounds. Rosmarinic acid has four hydroxyl groups attached to the phenyl ring; hence, it is expected that the rosemary extracts with high rosmarinic content are more effective antioxidants than those rich in carnosic acid, which only has two hydroxyl groups. The logic behind the previous statement lies in the fact that rosmarinic acid has more hydrogen atoms that can be donated to free radicals. In the study by Klančnik et al. [57], it was noted that the rosemary samples richer in rosmarinic acid displayed higher reducing powers and DPPH-scavenging potential than the samples rich in carnosic acid, while also displaying a lower activity in aqueous emulsion systems, such as the β-carotene bleaching assay. The following is due to the fact that in emulsions, polar antioxidants such as rosmarinic acid stay in the aqueous phase and offer limited protection against lipid oxidation. According to the authors, less polar compounds, such as carnosic acid, integrate into the lipid phase and interfaces, providing stronger antioxidant effects by stabilizing unsaturated fatty acids and preventing their deterioration. Nevertheless, it is important to consider that the overall antioxidant activity of plant extracts cannot be attributed to individual compounds alone. Phytochemicals can interact in complex ways: synergistically, additively, or antagonistically, depending on their relative concentrations and chemical compatibility. These interactions, in turn, are influenced by numerous factors, including the plant’s genetics, climate, and other environmental and soil conditions, as well as harvesting season, and the extraction method used [58]. Beyond its biological activities, *S. rosmarinus* extract has also been authorized by the European Commission as a technological antioxidant additive in pet animal nutrition (cats and dogs). This regulatory approval underscores its efficacy and safety in practical applications, suggesting potential relevance for other sectors where natural antioxidant strategies are sought. Integrating this perspective provides additional context for the technological potential of rosemary-derived compounds [59].

Furthermore, this study revealed that the rosemary extracts possess a high α-glucosidase inhibition potential compared to the standard substance, acarbose, especially the L3 samples. Although the alcoholic extracts showed an overall better activity when compared to the aqueous ones, the L3M and L3J aqueous extracts exerted a high inhibition of α-glucosidase activity. Our study of phytochemical composition of the investigated extracts revealed that they are abundant in carnosic, rosmarinic, and sagerinic acids, rosmanol, as well as (-)-20-Deoxocarnosol and carnosol (Tables S1 and S2). The hypoglycemic activity of carnosic and rosmarinic acids, as well as rosmanol, has been previously documented [60,61,62]; however, there is no clear evidence of the hypoglycemic activity of sagerinic acid so far. Our previous study reflected on the hypoglycemic activity of methanolic, ethanolic, and aqueous extracts of several Lamiaceae species from Serbia, including rosemary [63], and it is in accordance with our present study confirming that the alcoholic rosemary extracts have a higher hypoglycemic potential *in vitro* than the aqueous ones. Moreover, the dynamical model presented in the aforementioned study predicted that the rosemary extracts overall exert significant inhibitory potential for the human α-glucosidase, based on their phytochemical profile, suggesting potential application in the treatment of diabetes mellitus in humans. Actually, our previous work [63] demonstrated that the major constituents of the investigated Lamiaceae species bind efficiently to both the yeast as well as to the human enzyme, partly justifying the use of yeast glucosidase in these experiments. These findings support the existing data on the antioxidant properties of rosemary’s phenolic compounds that play a crucial role in improving insulin sensitivity and enhancing glucose uptake, while also triggering the liver to turn back to its normal homeostasis, which is vital for managing diabetes [12,64]. According to the American Diabetes Association’s Standards of Care 2025 [65], α-glucosidase inhibitors produce only modest reductions in HbA1c, a measure of average blood glucose over the preceding 2–3 months that serves as the gold-standard indicator of long-term glycemic management. This reflects on their primary effect on postprandial rather than overall glycemic control. As a result, they are not prioritized as frontline therapy compared with agents offering greater HbA1c lowering and cardio-renal benefits. On the other hand, glucosidase inhibition also has an ecological function in plants, serving as a defense mechanism against herbivorous insects by producing secondary metabolites that disrupt insect digestion, leading to mortality and reduced survival [66].

Patients with diabetes have an increased incidence of Alzheimer’s disease [12,67]. Hence, the information that rosemary extracts protect neurons from oxidative damage, inhibit amyloid-beta plaque formation, and enhance cognitive function by inhibiting enzymes related to neurodegeneration, such as acetylcholinesterase (AChE) and butyrylcholinesterase activity [4,12] is essential and should be thoroughly analyzed. In addition, it has been proven that the common denominator of neurodegenerative diseases is neuroinflammation, as well as various mitochondrial dysfunctions, which can also be associated with increased levels of oxidative and nitroxidative stress [68,69]. Hence, in this study, we aimed at revealing and comparing the antineurodegenerative potential of extracts of rosemary from different locations and harvested in different seasons by measuring the levels of AChE and TYR activities. Our results revealed that many of the tested extracts had higher enzyme-inhibitory activity compared to the standards used as positive controls. Regarding the AChE inhibition, rosemary from Lastva Grbaljska was the most potent, followed by the plant material from Bogatić, and the one from Belgrade. Furthermore, the aqueous extracts exhibited significantly higher anti-AChE activity, followed by the ethanolic and methanolic ones. On the other hand, in the TYR inhibition assay, the locality and the choice of solvent did not have a statistically significant impact. Overall, the results of this study are compelling, especially given that there is currently no cure for Alzheimer’s and Parkinson’s diseases. The accumulation of AChE and TYR contributes to the progression of these disorders, which are affecting an increasing number of people each year [70,71]. A recent study found that among the phytoconstituents of rosemary extracts, rosmanol, an abietane-type diterpene, exhibited a strong affinity for the active site of AChE, indicating it can be responsible for the high AChE-inhibitory activity of rosemary extracts [61,71]. On the other hand, Yaman et al. [72] reported that although the aqueous rosemary extract had a lower TPC and DPPH-inhibitory activity compared to the green tea extract tested in their study, it showed a higher DPPH-inhibitory activity when compared to the positive control, BHT. Furthermore, the aqueous rosemary extract demonstrated the highest TYR-inhibitory activity when compared to both the green tea extract and the positive control, ascorbic acid. The authors attributed this activity to the abundance of phenolic compounds, such as rosmarinic acid, in the extract; however, that was not the case in our study (Table S2). Unfortunately, the results of our chemical analysis did not single out the components responsible for the expressed antineurodegenerative activity; however oxygenated monoterpenoides seem to be more abundant in the L3 extracts when compared to the other two localities (Table S1). On the other hand, numerous *in vivo* and *in vitro* studies have demonstrated that rosemary extracts have potent ROS-scavenging, anti-inflammatory, and neuroprotective properties, which play a significant role in mitigating the development and progression of neurodegenerative disorders [60]. Finally, the combined immunomodulatory effects of rosemary highlight its potential as a therapeutic agent for diabetes and neurodegenerative diseases, warranting further studies to fully understand its efficacy.

Among its various health benefits, rosemary’s anticancer properties have garnered significant attention throughout the years. Its phytoconstituents offer several advantages in cancer treatment, including selective cytotoxicity, anti-proliferative, proapoptotic, and antiangiogenic effects, as well as synergistic effects with chemotherapy drugs. Moreover, rosemary constituents are known to inhibit the proliferation of cancer cells by inducing cell cycle arrest at various checkpoints (G0/G1, S, and G2/M phases), which prevents cancer cells from multiplying and spreading [7,14,15]. Bearing in mind that tumor growth and metastasis rely on the formation of new blood vessels (angiogenesis), it is noteworthy that carnosol and carnosic acid from rosemary are found to suppress angiogenesis by downregulating pro-angiogenic factors like vascular endothelial growth factor, thereby limiting tumor development [73].

Considering that the extracts of rosemary harvested in Lastva Grbaljska demonstrated the highest activity in previous chemical and biochemical-based assays, these extracts were selected for further, cell-based, investigation. Our results indicated that the tested L3 extracts are compatible with normal cells, while they expressed selective cytotoxicity towards HCT-116 colorectal cancer cells. This treat might come in handy when trying to minimize the side effects typically associated with conventional cancer treatments [16]. The L3J extract displayed the most significant cytotoxic effects, likely due to seasonal variations affecting the phytochemical content, which aligns with previous reports indicating that environmental factors such as light exposure, temperature, and harvesting time can significantly influence the biosynthesis of bioactive metabolites in rosemary [74]. Moreover, the extracts were more potent than the rosmarinic acid alone, which was expected due to the abundance of biologically active components revealed in this study, while the combined treatment of the rosmarinic acid and 5-FU showcased the strongest potential. According to the observed IC_50_ values for rosemary extracts and by comparing to the NCI referent value for cytotoxicity (<30 µg/mL), it can be concluded that the methanolic and ethanolic extracts induce strong cytotoxicity on HCT-116 colorectal cancer cells. Some authors suggest that higher concentrations of plant extracts, even up to 100 μg/mL, should be marked as effective [75]. Methanolic and ethanolic plant extracts, in particular, have been noted for their antitumor properties, as evidenced by their cytotoxicity (IC_50_ values) and selectivity. Furthermore, standardized rosemary extract and carnosic acid are reported to significantly increase the expression of Nrf2 in colon cells and inhibit an HCT116 xenograft tumor formation in mice [6]. Similar results were reported by other authors on HCT-116 and other colon cancer cell lines (SW480 and HT-29), emphasizing the critical role of extraction solvents in determining bioactive compound yield and efficacy [13,76,77]. Notably, a substantial number of rosemary extracts reported in the literature exhibit IC_50_ values below the 30 µg/mL threshold, which is generally regarded as indicative of meaningful cytotoxic potency for crude plant preparations. Published values typically range from 12.50 to 47.55 µg/mL across different extract types and cell lines [76,78,79,80]. Within this context, the IC_50_ value obtained in our study (<30 µg/mL) aligns closely with the more active examples reported in the literature. This comparison highlights that our extract not only falls well within the established cytotoxicity range for rosemary but also ranks among the more potent crude preparations, further underscoring its promising anticancer potential. Even relative to the positive control, the widely used cytostatic agent 5-fluorouracil (IC_50_ = 21.45 µg/mL for 24 h on HCT-116 [81], the cytotoxic effect of the rosemary extract remains remarkably pronounced, highlighting its substantial potency and reinforcing the promising nature of the observed IC_50_ values. The results of the anticancer potential in our study correlate with the obtained concentrations of phenolic content in the tested extracts, regarding the used solvent for extraction, as well as seasons.

Finally, the potential synergism between rosmarinic acid and the commercially used cytostatic for colorectal carcinoma, 5-FU, was evaluated in this study. In recent years, there has been increasing interest in combined treatments involving naturally obtained substances and chemotherapeutics, aiming to mitigate the wide range of side effects associated with high-dose chemotherapeutics, such as toxicity to normal cells and the development of cancer cell resistance [82]. Crude extracts and pure plant-derived compounds are frequently utilized in such co-treatments [83,84]. To investigate potential synergistic effects, rosmarinic acid was combined with 5-FU. The results demonstrated that the combined treatment with rosmarinic acid and 5-FU significantly reduced the viability of HCT-116 cells, exhibiting stronger cytotoxicity compared to the treatment with rosmarinic acid alone. The calculated CI values indicated strong synergistic interactions between these compounds, supporting their potential therapeutic application. This approach offers the possibility of reducing the required doses of chemotherapeutic agents while enhancing treatment efficacy, supporting the rationale for exploring phytochemical-drug interactions as a strategy to enhance cancer treatment outcomes. These findings align with previous reports suggesting that combining rosemary-derived compounds with chemotherapy drugs can improve treatment outcomes. For instance, Sirajudeen et al. [16] reported that carnosic and rosmarinic acids synergistically enhance the effectiveness of cancer therapy when used alongside traditional chemotherapeutic agents. Hence, isolating a single polyphenolic compound from rosemary extract may reduce the potential pharmacological effects across various cellular systems. However, Yan et al. [6] claim that utilizing standardized rosemary extract seems preferable to a single pure phytochemical, as it offers a diverse range of chemically related phytoconstituents. The abundance of phenolic compounds in rosemary actually underscores its biological significance and reinforces its potential as a valuable source of natural therapeutic agents.

The production of specialized secondary metabolites is crucial for plant survival, as these compounds help plants withstand environmental stresses while also offering valuable health benefits. Rosemary extracts are particularly notable for their bioactive properties, which are influenced by factors like geographic origin, season of harvest, and the solvents used during extraction. This highlights the importance of refining extraction methods and establishing standardized protocols. Climate conditions also play a key role in shaping the biosynthesis of these bioactive compounds, making it essential to study how seasonal and regional factors affect their production. While the conceptual framework of this study is not entirely novel [51], to the best of our knowledge, it represents the most comprehensive investigation of rosemary extracts to date. This study examines the effects of multiple factors on the detailed chemical composition and a wide spectrum of biological activities of rosemary extracts. Additionally, our research suggests that rosemary extracts enriched with rosmarinic acid could result in a more effective nutraceutical. By integrating these variables, the research provides a holistic understanding of how environmental and methodological conditions influence the bioactive potential of rosemary, offering valuable insights for its therapeutic and nutritional applications. However, monthly variations in phytochemicals across different ecological zones should be systematically studied in the future to understand the environmental and signaling factors influencing their synthesis and accumulation. Finally, this study highlighted the need for developing strategies to produce standardized plant material with maximum bioactivity, which is vital for unlocking the full therapeutic and dietary potential of these compounds.

## 4. Materials and Methods

### 4.1. Chemicals and Reagents

DPPH (2,2-diphenyl-1-picrylhydrazyl), acarbose, acetylcholine iodide, MTT (3-[4,5-dimethylthiazol-2-yl]-2,5-diphenyltetrazolium bromide), acetylcholinesterase from *Electrophorus electricus*, caffeic acid, DTNB (5,5′-ditio-bis(2-nitrobenzoic acid)), FBS (fetal bovine serum), Folin–Ciocalteu reagent, galantamine, gallic acid, iron(II) sulfate heptahydrate, iron(III) chloride, kojic acid, L-DOPA (3,4-dihydroxy-L-phenylalanine), *p*NPG (4-nitrophenyl β-D-glucopyranoside), quercetin, rosmarinic acid, TPTZ (2,4,6-tripyridil-s-triazin), tris base, tyrosinase from *Agaricus bisporus*, α-glucosidase from *Saccharomyces cerevisiae* type I, and β carotene were purchased from Merck, Darmstadt, Germany. Linoleic acid, Tween 40, and 5-fluorouracil (5-FU, Cat. No. 51-21-8) were obtained from Acros Organics, Belgium. Potassium ferricyanide(III) and trichloroacetic acid were purchased from Superlab, Belgrade, Serbia.

Acetonitrile (Fisher Scientific UK, Leicestershire, UK) and formic acid (Merck, Darmstadt, Germany) were of MS grade. Ultra-pure deionized water was generated using the Water Purification System (New Human Power I Integrate, Human Corporation, Seoul, Republic of Korea).

### 4.2. Plant Material

Rosemary leaves were harvested three times over nine months from three distinct locations: Belgrade and Bogatić in Serbia, and Lastva Grbaljska in Montenegro. The harvested material underwent air-drying and was subsequently packed in paper bags. It was then stored in a dry, dark room at room temperature until further processing. Voucher specimens have been placed in the Herbarium of the Institute of Botany and Botanical Garden “Jevremovac” at the University of Belgrade, Faculty of Biology. Detailed information regarding the plant material can be found in Table S4.

### 4.3. Extracts Preparation and Determination of Extraction Yield

Extracts were prepared from dry leaves by pulverizing the material in a laboratory blender, resulting in fragments measuring 2–6 mm in size. Subsequently, 3 g of diced plant material was weighed and combined with 30 mL of the respective solvent (70% methanol, 70% ethanol, and hot distilled water). A total of 27 extracts were prepared, considering the collection of plant material from three distinct locations, harvested on three occasions, and extracted using three varied solvents. The mixture of solvent and plant material underwent ultrasonic exposure (30 °C) for the initial and final hour of the 24 h maceration process.

Following maceration, the extracts were sieved through filter paper, and the surplus solvent was eliminated by a rotary vacuum evaporator (Büchi rotavapor R-114, BÜCHI Labortechnik AG, Flawil, Switzerland). The resultant dry extracts were refrigerated at +4 °C until subsequent experiments.

The dry extract was weighed, and the extract yield was determined using the following equation:

[m/M] × 100%,

where m represents the mass of the dry extract (in grams), and M represents the mass of dry plant material used for the extraction (3 g).

### 4.4. Determination of Phytochemical Content

All assessments of the phytochemical content for rosemary extracts were conducted in three concentrations: 100, 250, and 500 μg/mL, and the absorbances were measured using a Perkin Elmer Lambda Bio UV/VIS spectrophotometer (PerkinElmer Inc., Shelton, CT, USA).

The total phenolic content (TPC) and total flavonoid content (TFC) were assessed following previously established methods [85,86]. TPC was measured at 740 nm and determined using the gallic acid curve equation. The results were expressed as milligrams of gallic acid equivalents (GAE) per gram of dry extract. Similarly, TFC was measured at 415 nm and determined using the quercetin curve equation. The results were expressed as milligrams of quercetin equivalents (QE) per gram of dry extract.

The quantification of phenolic acid content (PAC) and flavonol content (FC) was conducted according to previously described methods [87], at wavelengths of 490 nm and 440 nm, respectively. PAC was expressed as milligrams of caffeic acid equivalents (CAE), while FC was expressed as milligrams of QE per gram of dry extract.

All findings were presented as means ± standard deviations (SD), averaged from three measurements.

### 4.5. LC/MS Metabolite Identification

LC-HRMS/MS (Thermo Scientific™ Vanquish™ Core HPLC system coupled to the Orbitrap Exploris 120 mass spectrometer, San Jose, CA, USA) was used to determine the metabolic profile of the extracts. All LC/MS settings were previously described in detail by Stojković et al. [88]. LC/MS data was evaluated using R Studio (version 2023.09.1, build 494) software. Peak picking was performed using the enviPick R package, and peak correspondence across samples was performed using the density method available in the xcms R package [89].

### 4.6. GC/MS Analysis

Volatile terpenoids present in 27 rosemary samples were profiled using an Agilent 8890 gas chromatograph (GC) coupled with a Mass Selective Detector (5977B GC/MSD, Agilent Technologies, Santa Clara, CA, USA) and connected to an automated sample extraction and enrichment platform (Centri^®^, Markes International Ltd., Bridgend, UK). Compounds were chromatographically separated for 21 min on an HP 5MScolumn (30 m × 0.25 mm, 0.25 µm film thickness; Agilent Technologies, Santa Clara, CA, USA), using helium (99.999%, The Linde Group, Dublin, Ireland) as a carrier gas at a flow rate of 1.6 mL min^−1^. Settings of the mass spectrometer were as follows: the transfer line temperature of 280 °C, and the detector temperature of 270 °C. Mass spectra were acquired in the positive EI mode (+70 eV), and the EI source temperature was set to 280 °C. Column temperature was linearly programmed from 40 to 300 °C at a rate of 20 °C min^−1^ and held isothermally at 240 °C for the subsequent 5 min. The analyses were performed in the SCAN mode, tracking the compounds within the range from 45 to 500 amu. Identification of constituents in the rosemary samples was performed by comparing their mass spectra and retention times with those of the respective standards (Terpene Mix B, CRM40937, purchased from Supelco Park, 595 North Harrison Road, Bellefonte, PA 16823, USA), as well as by comparison with the NIST05 library.

### 4.7. Determination of the Antioxidant Activity

All evaluations of the antioxidant activity of rosemary extracts were conducted in three concentrations: 100, 250, and 500 μg/mL, and the absorbances were measured using a Perkin Elmer Lambda Bio UV/VIS spectrophotometer.

DPPH assay was assessed following a previously established protocol by Blois [90], using ascorbic acid, BHA, and BHT as positive controls, and the absorbances were measured at a wavelength of 517 nm. The results were presented as percentages of DPPH radical inhibition.

FRAP assay was evaluated according to Benzie and Strain [53] using ascorbic acid, BHA, and BHT as positive controls, and the absorbances were measured at 593 nm. The results were determined using the curve equation for the solution of ferrous sulfate heptahydrate (0.2–1.6 mmol/L) and expressed as micrograms of ferrous sulfate heptahydrate per gram of dry extract.

The total reducing power (TRP) assay was assessed following the protocols by Oyaizu [91] and Tusevski et al. [92]. The absorbances were measured at 700 nm, while the results were presented as micromoles of ascorbic acid equivalents (AAE) per gram of dry extract.

The β-carotene bleaching assay was assessed based on a previously described procedure by Dapkevicius et al. [93]. Ascorbic acid, BHA, and BHT were used as positive controls. The absorbances were measured at 490 nm, and the results were presented as percentages of β-carotene bleaching inhibition.

All findings were presented as means ± SD, averaged from three measurements.

### 4.8. Determination of Enzyme Inhibition

The α-glucosidase inhibitory activity of the extracts was tested at concentrations ranging from 100 to 2000 μg/mL, while their AChE and TYR inhibitory activities were assessed at 100, 250, and 500 μg/mL. These analyses were carried out using a Multiskan Sky Thermo Scientific microplate reader.

The inhibition of α-glucosidase activity was evaluated following the protocol by Wan et al. [94] using acarbose as a positive control. In the sample wells (S) of the 96-microtiter plate, 120 μL of extract at the appropriate concentrations and 20 μL of enzyme solution (0.5 U/mL) prepared in potassium phosphate buffer (0.1 M, pH 6.8) were added. After a 5 min incubation at 37 °C, 20 μL of 5 mM *p*NPG was added and the mixture was incubated for 20 min at 37 °C. The reaction was stopped by adding 80 μL of 0.2 M sodium carbonate in buffer. The negative control (C) contained buffer instead of the sample, while the blank control (B) contained all components except the enzyme, which was replaced with buffer. All measurements were performed in triplicate. Absorbance was measured at 405 nm using a Multiskan Sky microplate reader (Thermo Scientific, Vantaa, Finland). The percentage of α-glucosidase inhibition (%) was calculated using the following equation:

α-Glucosidase inhibition (%) = [C − (S − B)]/C × 100.

Furthermore, the AChE inhibition assay was conducted following the procedure of Ellman et al. [95] using galantamine as a positive control. The absorbances were measured at 412 nm, while the results were expressed as percentages of inhibition of AChE activity. The TYR inhibition assay was assessed according to the protocol by Masuda et al. [96] using kojic acid as a positive control. All absorbances were measured at 475 nm, while the results were expressed as percentages of inhibition of TYR activity.

All findings were presented as means ± SD, averaged from three measurements.

### 4.9. Determination of Anticancer Potential of the Extracts and Rosmarinic Acid

In this study, the extracts and 5-FU were initially dissolved in DMSO, followed by the preparation of the final working concentrations in cell culture medium from the stock solution. The final DMSO concentration in the highest applied concentration was 0.5%.

The HCT-116 colorectal cancer cell line, sourced from the American Type Culture Collection, and HaCaT normal human keratinocytes from Cell Lines Service (Eppelheim, Germany) were cultured in DMEM enriched with 10% FBS, 100 units/mL penicillin, and 100 μg/mL streptomycin under optimal conditions as per established protocols [97]. Once the cells reached 70–90% confluence after several passages, they were seeded for the MTT assay.

The cytotoxicity of rosemary extracts was assessed using the MTT assay [98]. The colorectal cancer HCT-116 and HaCaT cells, as normal control, were treated with methanolic, ethanolic, and aqueous rosemary extracts, harvested in Lastva Grbaljska, Montenegro, across three seasons (samples L3N, L3M, and L3J) and tested at various concentrations (1, 10, 50, 100, 250, and 500 μg/mL), for 24 and 72 h. The rosmarinic acid (0.1, 1, 5, 10, 50, and 250 μg/mL) was also tested, as one of the dominant compounds of the rosemary extracts. Untreated cells served as negative controls. After the treatment period, MTT (5 mg/mL in PBS) was added to each well, and the plates were incubated at 37 °C in a 5% CO_2_ atmosphere for 2–4 h. The resulting formazan crystals were dissolved in DMSO, and absorbance was read at 550 nm using a microplate reader. Cell viability was calculated as the absorbance ratio of treated samples to controls, expressed as a percentage of viable cells. The IC_50_ values were calculated in the CalcuSyn software program from cell viability curves.

#### Determination of Synergistic, Additive, and Antagonistic Effects of Rosmarinic Acid and 5-FU

Potential synergistic, additive, or antagonistic effects of rosmarinic acid and 5-FU, a known chemotherapeutic used for colorectal cancer, were evaluated by comparing the effects (inhibition of cell growth) obtained in the individual treatment with the cell growth inhibition from a combination of treatments. Rosmarinic acid was applied in the same range of concentrations as in the MTT assay (0.1, 1, 5, 10, 50, and 250 μg/mL), and 5-FU was added in a concentration of 1 µg/mL for all combination samples. Standard MTT protocol was conducted. The value of the combination index (CI), as a parameter for interactions, was calculated in the software program CalcuSyn. According to Chou and Talalay [99], values less than 0.8 indicate synergistic interactions, values between 0.8 and 1 suggest additive effects, and values greater than 1 indicate antagonistic interactions.

### 4.10. Statistical Analysis

All of the experimental measurements were carried out in triplicate, and the results are expressed as the average of three measurements ± SD. The analysis of variance (single-factor ANOVA) and Tukey’s post hoc test were performed using the Statistical Package for Social Sciences program (IBM SPSS Statistics for Windows, Version 25.0, IBM Corporation, Armonk, NY, USA), to test the significance of the differences among the mean values. The minimum probability value taken as statistically significant was *p* < 0.05. Integrated Biomarker Response (IBR) values were calculated, and the results were standardized according to Beliaeff and Burgeot [100].

## 5. Conclusions

Using *Salvia rosmarinus* as a model species, we examined samples (i) collected from three locations—Belgrade and Bogatić (Serbia) and Lastva Grbaljska (Montenegro); (ii) harvested in different seasons—November, March, and July; and (iii) extracted using different solvents—methanol, ethanol, and hot distilled water. The results revealed pronounced antioxidant and enzyme-inhibitory activities, strongly dependent on location, harvest time, and solvent choice. The IBR analysis highlighted the samples from Lastva Grbaljska as the ones with the highest bioactivity, which is the reason they were subsequently selected to be evaluated for antitumor activities. The Lastva Grbaljska extracts demonstrated selective cytotoxicity toward HCT-116 colorectal cancer cells while protecting normal cells. Furthermore, co-treatment with rosmarinic acid and the chemotherapeutic agent 5-fluorouracil enhanced the latter’s efficacy, suggesting the potential for dose reduction and improved therapeutic outcomes.

Comprehensive GC/MS and LC/MS analyses of rosemary samples identified a wide spectrum of volatile and non-volatile constituents, reflecting the plant’s remarkable chemical diversity. GC/MS profiling revealed 28 terpenoid compounds, including mono-, sesqui-, di-, and triterpenes, with ethanol emerging as the most efficient solvent for extracting the majority of terpenoid classes. Actually, triterpenes (β-amyrin and α-amyrin) were only found in ethanolic and aqueous extracts. In parallel, LC/MS analysis identified 92 distinct metabolites encompassing phenolic acids, flavonoids, terpenoids, and other minor compounds, 28 of which are identified in *Rosmarinus* sp. extracts for the first time, thereby providing a detailed chemical fingerprint of rosemary extracts. In general, it was observed that some phenolic acids (mainly rosmarinic acid derivatives), some flavonoid glycosides, and diterpenes are the main contributors to the regional chemical differentiation between samples from Serbia and Montenegro, as they were observed to be more represented in samples from Montenegro. The combined analytical data highlight the complex phytochemical composition of rosemary and underscore its considerable potential as a source of bioactive and therapeutically valuable natural compounds.

Although the conceptual framework of this study is not entirely new, it represents, to the best of our knowledge, the most comprehensive investigation of rosemary extracts to date. Notably, several assays were performed for the first time on extracts obtained from different locations, seasons, and solvents, providing novel insights into the factors shaping the chemical composition and bioactivity of this species. The findings reported in this study underscore the necessity of understanding how environmental variability shapes the biosynthesis of specialized metabolites and, consequently, the biological efficacy of plant-derived products. Future research should focus on assessing the resilience of rosemary under diverse environmental conditions and developing strategies to standardize extracts with optimized and consistent metabolite profiles. Such an integrative approach will not only deepen our understanding of plant biochemical adaptation but also enhance the utilization of rosemary in therapeutic, nutraceutical, and functional food applications.

## Figures and Tables

**Table 1:** Yield (%) of the examined methanolic, ethanolic, and aqueous rosemary extracts from three localities (L1–3), and three seasons (November, N, March, M, and July, J).

Plant Species	Locality (L)	Harvesting Month	Abbreviations	Methanolic Extract	Ethanolic Extract	Aqueous Extract
*Salvia rosmarinus* Spenn.	Belgrade (1)	November (N)	L1N	13.07	8.77	14.43
		March (M)	L1M	9.60	8.93	12.90
		July (J)	L1J	9.20	9.17	11.47
	Bogatić (2)	November (N)	L2N	9.10	9.17	13.67
		March (M)	L2M	9.67	8.97	14.73
		July (J)	L2J	9.60	8.80	13.63
	Lastva Grbaljska (3)	November (N)	L3N	11.40	13.47	12.97
		March (M)	L3M	12.87	15.90	18.70
		July (J)	L3J	10.93	14.37	14.77

**Table 2:** Total phenolic (TPC), phenolic acid (PAC), flavonoid (TFC), and flavonol (FC) contents of the examined methanolic, ethanolic, and aqueous rosemary extracts from three localities (L1–3), and three seasons (November, N, March, M, and July, J).

Plant Material	Extract	TPC (mg GAE/g)	PAC (mg CAE/g)	TFC (mg QE/g)	FC (mg QE/g)
L1N	methanolic	93.23 ± 6.17 ^chi^	176.41 ± 7.14 ^bchi^	19.29 ± 1.36 ^bci^	5.48 ± 0.26 ^bi^
	ethanolic	78.65 ± 4.22 ^cgi^	130.48 ± 8.98 ^bcgi^	16.12 ± 0.96 ^bc^	4.98 ± 0.46 ^bi^
	aqueous	65.61 ± 1.52 ^cgh^	93.07 ± 6.79 ^bcgh^	14.35 ± 0.63 ^bcg^	2.03 ± 0.06 ^bgh^
L1M	methanolic	117.63 ± 4.34 ^cfhi^	168.26 ± 4.63 ^bcfhi^	25.51 ± 0.73 ^bcfi^	4.42 ± 0.15 ^bi^
	ethanolic	93.50 ± 5.61 ^cfgi^	107.89 ± 2.94 ^bcfgi^	18.39 ± 0.75 ^bcf^	2.15 ± 0.21 ^bi^
	aqueous	74.74 ± 5.59 ^cfgh^	74.93 ± 1.70 ^bcfgh^	16.93 ± 1.29 ^bcfg^	0.86 ± 0.09 ^bgh^
L1J	methanolic	73.27 ± 1.96 ^cehi^	52.70 ± 5.59 ^bcehi^	13.33 ± 0.39 ^bcei^	3.63 ± 0.32 ^bi^
	ethanolic	60.55 ± 3.62 ^cegi^	67.89 ± 4.01 ^bcegi^	13.64 ± 0.66 ^bce^	5.06 ± 0.35 ^bi^
	aqueous	60.19 ± 5.84 ^cegh^	58.63 ± 6.42 ^bcegh^	10.60 ± 1.83 ^bceg^	1.73 ± 0.12 ^bgh^
L2N	methanolic	89.84 ± 3.53 ^chi^	87.52 ± 1.28 ^achi^	14.45 ± 0.65 ^aci^	1.92 ± 0.10 ^aci^
	ethanolic	65.38 ± 5.97 ^cgi^	64.93 ± 5.48 ^acgi^	11.03 ± 0.55 ^ac^	1.67 ± 0.19 ^aci^
	aqueous	42.87 ± 1.98 ^cgh^	28.26 ± 1.28 ^acgh^	9.10 ± 0.48 ^acg^	0.80 ± 0.09 ^acgh^
L2M	methanolic	107.62 ± 1.43 ^cfhi^	107.52 ± 3.39 ^acfhi^	14.23 ± 0.52 ^acfi^	0.99 ± 0.30 ^aci^
	ethanolic	79.93 ± 3.52 ^cfgi^	84.56 ± 2.22 ^acfgi^	10.97 ± 0.25 ^acf^	2.02 ± 0.49 ^aci^
	aqueous	73.01 ± 4.96 ^cfgh^	124.93 ± 6.79 ^acfgh^	14.63 ± 1.03 ^acfg^	1.19 ± 0.26 ^acgh^
L2J	methanolic	93.66 ± 1.83 ^cehi^	93.81 ± 7.80 ^acehi^	21.59 ± 0.57 ^acei^	4.66 ± 0.41 ^aci^
	ethanolic	59.47 ± 4.22 ^cegi^	41.22 ± 2.94 ^acegi^	11.87 ± 0.90 ^ace^	2.84 ± 0.32 ^aci^
	aqueous	30.38 ± 0.93 ^cegh^	21.22 ± 4.01 ^acegh^	5.44 ± 0.78 ^aceg^	ND ^acgh^
L3N	methanolic	127.42 ± 6.86 ^abhi^	189.00 ± 6.19 ^abhi^	23.18 ± 0.09 ^abi^	4.94 ± 0.12 ^bi^
	ethanolic	116.33 ± 5.28 ^abgi^	148.26 ± 2.57 ^abgi^	22.83 ± 1.25 ^ab^	5.73 ± 0.31 ^bi^
	aqueous	100.80 ± 4.28 ^abgh^	122.33 ± 2.22 ^abgh^	18.95 ± 1.59 ^abg^	1.03 ± 0.15 ^bgh^
L3M	methanolic	134.60 ± 4.93 ^abfhi^	211.96 ± 4.49 ^abfhi^	24.11 ± 1.37 ^abfi^	4.00 ± 0.49 ^bi^
	ethanolic	128.92 ± 0.98 ^abfgi^	176.04 ± 1.70 ^abfgi^	25.54 ± 2.00 ^abf^	3.98 ± 0.22 ^bi^
	aqueous	80.74 ± 2.13 ^abfgh^	56.41 ± 6.12 ^abfgh^	22.68 ± 0.23 ^abfg^	4.60 ± 0.29 ^bgh^
L3J	methanolic	113.95 ± 4.82 ^abehi^	186.78 ± 7.78 ^abehi^	21.13 ± 1.40 ^abei^	4.83 ± 0.45 ^bi^
	ethanolic	109.77 ± 3.38 ^abegi^	139.74 ± 8.19 ^abegi^	18.70 ± 0.89 ^abe^	4.35 ± 0.23 ^bi^
	aqueous	88.96 ± 3.03 ^abegh^	100.85 ± 1.70 ^abegh^	24.76 ± 1.15 ^abeg^	3.50 ± 0.64 ^bgh^

Within one column, mean values with different superscript letters differ significantly (single-factor ANOVA, Tukey post hoc test, *p* < 0.05): a—vs. Locality 1; b—vs. Locality 2; c—vs. Locality 3; e—vs. March; f—vs. July; g—vs. methanolic extracts; h—vs. ethanolic extracts; i—vs. aqueous extracts. ND—not detected.

**Table 3:** The antioxidant activity of the examined methanolic, ethanolic, and aqueous rosemary extracts from three localities (L1–3), and three seasons (November, N, March, M, and July, J) was assessed through DPPH, FRAP, TRP, and β-carotene bleaching assays.

Plant Material	Extract	DPPH (%)	FRAP (μg FeSO_4_ × 7H_2_O/g)	TRP (μM AAE/g)	β-Carotene Bleaching (%)
L1N	methanolic	76.75 ± 0.59 ^cix–z^	564.74 ± 45.96 ^chixz^	752.39 ± 9.64 ^chi^	63.96 ± 2.64 ^hiz^
	ethanolic	67.35 ± 1.77 ^cix–z^	349.52 ± 34.33 ^cgx–z^	503.91 ± 14.88 ^cg^	69.35 ± 5.58 ^giz^
	aqueous	54.19 ± 0.65 ^cghz^	361.33 ± 18.39 ^cgx–z^	355.12 ± 12.38 ^cg^	50.96 ± 4.24 ^ghxz^
L1M	methanolic	75.68 ± 1.91 ^cix–z^	609.80 ± 27.66 ^chixz^	893.30 ± 2.29 ^chi^	64.47 ± 5.49 ^hiz^
	ethanolic	71.27 ± 3.47 ^cix–z^	429.57 ± 24.21 ^cgx–z^	564.21 ± 19.21 ^cg^	65.33 ± 2.83 ^giz^
	aqueous	60.12 ± 0.69 ^cghyz^	400.26 ± 21.12 ^cgx–z^	493.91 ± 16.66 ^cg^	45.21 ± 6.20 ^ghz^
L1J	methanolic	55.06 ± 2.68 ^ciz^	369.20 ± 37.72 ^chix–z^	544.21 ± 2.92 ^chi^	63.62 ± 5.59 ^hiz^
	ethanolic	49.09 ± 0.64 ^cix–z^	272.97 ± 34.72 ^cgx–z^	464.52 ± 21.05 ^cg^	74.33 ± 2.02 ^gix–z^
	aqueous	48.78 ± 0.80 ^cghx–z^	335.08 ± 22.82 ^cgx–z^	376.03 ± 30.35 ^cg^	39.27 ± 6.81 ^ghxz^
L2N	methanolic	75.61 ± 1.75 ^cix–z^	488.63 ± 11.84 ^chix–z^	563.91 ± 24.71 ^chi^	71.91 ± 4.58 ^hiz^
	ethanolic	57.84 ± 1.95 ^ciz^	290.46 ± 8.33 ^cgx–z^	377.85 ± 5.17 ^cg^	70.31 ± 4.39 ^giz^
	aqueous	33.37 ± 0.59 ^cghx–z^	246.28 ± 4.97 ^cgx–z^	267.85 ± 8.45 ^cg^	50.38 ± 5.98 ^ghz^
L2M	methanolic	73.55 ± 2.86 ^cix–z^	570.87 ± 46.44 ^chixz^	720.27 ± 16.59 ^chi^	62.77 ± 3.46 ^hiz^
	ethanolic	66.67 ± 1.32 ^cix–z^	354.33 ± 22.73 ^cgx–z^	606.33 ± 13.25 ^cg^	66.28 ± 3.74 ^giz^
	aqueous	59.70 ± 1.10 ^cghyz^	450.57 ± 20.93 ^cgx–z^	668.76 ± 8.25 ^cg^	45.79 ± 3.83 ^ghx–z^
L2J	methanolic	78.65 ± 0.60 ^cix–z^	579.18 ± 36.28 ^chixz^	641.79 ± 25.72 ^chi^	63.96 ± 1.02 ^hiz^
	ethanolic	48.44 ± 1.65 ^cixz^	287.84 ± 27.66 ^cgx–z^	366.33 ± 16.50 ^cg^	73.37 ± 4.89 ^giz^
	aqueous	25.80 ± 1.20 ^cghx–z^	207.35 ± 6.01 ^cgx–z^	267.85 ± 14.70 ^cg^	43.68 ± 2.87 ^ghx–z^
L3N	methanolic	89.99 ± 0.83 ^abixy^	873.58 ± 36.80 ^abhix–z^	1028.15 ± 16.52 ^abhi^	64.97 ± 1.52 ^hiz^
	ethanolic	87.86 ± 1.75 ^abixy^	554.24 ± 11.16 ^abgxz^	830.88 ± 0.52 ^abg^	66.67 ± 3.77 ^giz^
	aqueous	83.75 ± 0.70 ^abghx–z^	663.60 ± 39.86 ^abgxz^	719.36 ± 4.17 ^abg^	44.25 ± 4.34 ^ghx–z^
L3M	methanolic	85.77 ± 0.63 ^abix–z^	822.40 ± 6.47 ^abhix–z^	1182.39 ± 1.05 ^abhi^	62.77 ± 4.43 ^hiz^
	ethanolic	88.43 ± 0.37 ^abix–z^	654.86 ± 72.27 ^abgxz^	877.85 ± 4.30 ^abg^	72.41 ± 2.07 ^gix–z^
	aqueous	55.25 ± 1.54 ^abghyz^	388.89 ± 12.47 ^abgx–z^	519.36 ± 5.68 ^abg^	57.66 ± 5.43 ^ghz^
L3J	methanolic	77.74 ± 1.19 ^abix–z^	716.54 ± 38.46 ^abhix–z^	1097.55 ± 16.44 ^abhi^	60.07 ± 4.82 ^hiz^
	ethanolic	80.48 ± 0.64 ^abix–z^	562.99 ± 49.54 ^abgxz^	870.27 ± 11.02 ^abg^	67.05 ± 2.18 ^giz^
	aqueous	69.79 ± 3.67 ^abghx–z^	531.06 ± 7.23 ^abgx–z^	710.88 ± 9.11 ^abg^	53.45 ± 1.89 ^ghxz^
BHA		55.43 ± 1.38 ^c^	1596.33 ± 0.69 ^a–i^	NT	64.47 ± 0.31 ^i^
BHT		52.01 ± 0.53 ^cg^	559.00 ± 0.96	NT	59.12 ± 0.83 ^hi^
Ascorbic acid		89.70 ± 0.10 ^acfi^	1021.00 ± 2.2 ^a–i^	NT	6.92 ± 0.96 ^a–i^

Within one column, mean values with different superscript letters differ significantly (single-factor ANOVA, Tukey post hoc test, *p* < 0.05): a—vs. Belgrade; b—vs. Bogatić; c—vs. Lastva Grbaljska; d—vs. November; e—vs. March; f—vs. July; g—vs. methanolic extract; h—vs. ethanolic extract; i—vs. aqueous extract. Within one column, mean values with different superscript letters differ significantly (independent-sample t-test, *p* ≤ 0.05): x—vs. BHA; y—vs. BHT; z—vs. ascorbic acid. NT—not tested.

**Table 4:** The inhibition of α-glucosidase, acetylcholinesterase (AChE), and tyrosinase (TYR) activities of the examined methanolic, ethanolic, and aqueous rosemary extracts from three localities (L1–3), and three seasons (November, N, March, M, and July, J).

Plant Material	Extract	α-Glucosidase (%)	AChE (%)	TYR (%)
L1N	methanolic	57.91 ± 7.52 ^ci^	22.58 ± 6.31 ^bcix^	49.52 ± 1.49 ^cx^
	ethanolic	72.64 ± 7.11 ^cix^	38.13 ± 5.34 ^bcx^	57.62 ± 6.44 ^cx^
	aqueous	4.67 ± 1.70 ^cghx^	41.33 ± 3.64 ^bcgx^	49.29 ± 3.78 ^c^
L1M	methanolic	76.34 ± 2.51 ^cix^	41.96 ± 2.95 ^bcix^	53.57 ± 0.71 ^cx^
	ethanolic	60.54 ± 4.67 ^ci^	43.61 ± 7.78 ^bcx^	58.33 ± 4.06 ^cx^
	aqueous	30.33 ± 4.29 ^cghx^	42.69 ± 9.47 ^bcgx^	48.10 ± 2.70 ^c^
L1J	methanolic	40.31 ± 4.17 ^cix^	60.81 ± 7.80 ^bci^	45.27 ± 2.15 ^c^
	ethanolic	59.44 ± 6.37 ^ci^	60.57 ± 7.35 ^bc^	46.02 ± 6.35 ^c^
	aqueous	7.93 ± 1.12 ^cghx^	86.24 ± 6.62 ^bcgx^	42.29 ± 2.28 ^c^
L2N	methanolic	80.59 ± 3.04 ^cix^	60.55 ± 6.90 ^ai^	52.49 ± 1.14 ^cx^
	ethanolic	91.90 ± 3.66 ^cix^	70.25 ± 9.38 ^a^	42.29 ± 4.31 ^c^
	aqueous	11.70 ± 1.32 ^cghx^	74.83 ± 9.32 ^ag^	39.55 ± 4.66 ^c^
L2M	methanolic	65.01 ± 4.25 ^ci^	38.66 ± 8.64 ^aix^	51.85 ± 1.21 ^cx^
	ethanolic	44.72 ± 5.98 ^cix^	64.52 ± 8.02 ^a^	54.23 ± 0.92 ^cx^
	aqueous	ND ^cghx^	78.47 ± 3.62 ^agx^	47.62 ± 2.10 ^c^
L2J	methanolic	57.69 ± 1.77 ^ci^	56.72 ± 7.75 ^ai^	47.09 ± 3.21 ^c^
	ethanolic	86.36 ± 3.52 ^cix^	59.33 ± 6.03 ^a^	42.86 ± 0.79 ^c^
	aqueous	7.64 ± 1.78 ^cghx^	86.30 ± 8.21 ^agx^	37.04 ± 0.46 ^cx^
L3N	methanolic	88.67 ± 1.45 ^abix^	79.22 ± 3.84 ^aix^	60.63 ± 3.03 ^abx^
	ethanolic	98.64 ± 0.76 ^abix^	80.30 ± 9.08 ^ax^	52.30 ± 1.00 ^abx^
	aqueous	22.45 ± 1.23 ^abghx^	78.94 ± 2.82 ^agx^	54.89 ± 2.17 ^abx^
L3M	methanolic	79.34 ± 4.53 ^abix^	73.72 ± 1.94 ^aix^	52.59 ± 2.28 ^abx^
	ethanolic	90.45 ± 1.22 ^abix^	84.84 ± 2.67 ^ax^	50.86 ± 1.72 ^abx^
	aqueous	98.17 ± 0.25 ^abghx^	80.20 ± 1.67 ^agx^	54.31 ± 0.86 ^abx^
L3J	methanolic	70.86 ± 1.07 ^abix^	42.86 ± 6.64 ^aix^	59.71 ± 5.10 ^abx^
	ethanolic	81.35 ± 4.09 ^abix^	68.21 ± 4.36 ^a^	58.26 ± 2.30 ^abx^
	aqueous	98.10 ± 1.56 ^abghx^	73.14 ± 6.01 ^ag^	58.26 ± 0.87 ^abx^
Acarbose		58.05 ± 1.00	NT	NT
Galantamine		NT	63.21 ± 2.64	NT
Kojic acid		NT	NT	45.59 ± 1.12 ^c^

Within one column, mean values with different superscript letters differ significantly (single-factor ANO-VA, Tukey post hoc test, *p* < 0.05): a—vs. Belgrade; b—vs. Bogatić; c—vs. Lastva Grbljanska; g—vs. methanolic extract; h—vs. ethanolic extract; i—vs. aqueous extract. Within one column, mean values with “x” differ significantly (independent-sample *t*-test, *p* ≤ 0.05): vs. acarbose; vs. galantamine; vs. kojic acid. ND—not detected; NT—not tested.

**Table 5:** The cytotoxicity of rosemary extracts on human colorectal cancer cells, HCT-116, and normal human keratinocytes (HaCat), expressed as IC_50_ values (µg/mL).

Plant Material	Extract	HCT-116		HaCat	
		24 h	72 h	24 h	72 h
L3N	methanolic	39.43 ± 0.66	12.27 ± 0.61	>500	>500
	ethanolic	37.90 ± 0.99	15.65 ± 0.01	>500	>500
	aqueous	>500	481.30 ± 2.36	>500	>500
L3M	methanolic	46.94 ± 0.41	18.57 ± 0.91	>500	>500
	ethanolic	56.30 ± 2.21	62.64 ± 2.32	>500	>500
	aqueous	>500	492.12 ± 2.37	>500	>500
L3J	methanolic	13.08 ± 0.52	18.69 ± 2.45	>500	>500
	ethanolic	21.25 ± 1.12	22.46 ± 1.05	>500	>500
	aqueous	215.97 ± 4.12	311.71 ± 2.65	>500	>500
Rosmarinic acid		64.86 ± 2.14	57.84 ± 3.15	>250	>250
Combined treatment of rosmarinic acid and 5-FU (1 µg/mL)		4.18 ± 0.021	0.17 ± 0.01	NA	NA

NA—not assessed.

**Table 6:** The combined treatment and interaction between rosmarinic acid and 5-FU of HCT-116 cells.

Treatments	Exposure Time	Cell Viability (%)	Interaction	CI *
5-FU (1 µg/L)	24 h 72 h	84.35 58.58	- -	- -
Rosmarinic acid (0.1 µg/mL)	24 h 72 h	100 94.70	- -	- -
Rosmarinic acid (1 µg/mL)	24 h 72 h	100 98.41	- -	- -
Rosmarinic acid (5 µg/mL)	24 h 72 h	99.09 93.42	- -	- -
Rosmarinic acid (10 µg/mL)	24 h 72 h	82.55 88.87	- -	- -
Rosmarinic acid (50 µg/mL)	24 h 72 h	48.18 81.03	- -	- -
Rosmarinic acid (250 µg/mL)	24 h 72 h	11.14 3.29	- -	- -
Rosmarinic acid (0.1 µg/mL) + 5-FU (1 µg/mL)	24 h 72 h	43.21 38.21	Synergistic Synergistic	0.258 0.246
Rosmarinic acid (1 µg/mL) + 5-FU (1 µg/mL)	24 h 72 h	57.18 36.99	Synergistic Synergistic	0.409 0.270
Rosmarinic acid (5 µg/mL) + 5-FU (1 µg/mL)	24 h 72 h	59.30 35.49	Synergistic Synergistic	0.545 0.248
Rosmarinic acid (10 µg/mL) + 5-FU (1 µg/mL)	24 h 72 h	57.37 33.40	Synergistic Synergistic	0.644 0.275
Rosmarinic acid (50 µg/mL) + 5-FU (1 µg/mL)	24 h 72 h	8.59 20.51	Synergistic Synergistic	0.240 0.225
Rosmarinic acid (250 µg/mL) + 5-FU (1 µg/mL)	24 h 72 h	12.39 3.09	Synergistic Synergistic	0.576 0.022

* Combination index (CI) indicates synergism (<0.8), additive effects (0.8–1) or antagonistic interactions (>1), calculated in the software program CalcuSyn (Version 2.0).

## Data Availability

The original contributions presented in this study are included in the article/Supplementary Materials. Further inquiries can be directed to the corresponding authors.

## Supplementary Materials

The following supporting information can be downloaded at: https://www.mdpi.com/article/10.3390/plants15010001/s1, Figure S1: Radar plots generated from IBR analysis illustrating the overall bioactivity of methanolic, ethanolic, and aqueous extracts of *Salvia rosmarinus* harvested from three locations (L1—Belgrade, Serbia; L2—Bogatić, Serbia; L3—Lastva Grbaljska, Montenegro) and across three seasons (N—November; M—March; J—July). The plots are based on data obtained from four antioxidant assays: DPPH, FRAP, total reducing power (TRP), and β-carotene bleaching, as well as three enzyme-inhibitory assays: α-glucosidase, acetylcholinesterase (AChE), and tyrosinase (TYR) inhibition. The area enclosed by each radar plot represents the overall tested bioactivity of the sample: the larger the area, the higher the overall biological activity; Figure S2: The cytotoxicity (y-axis) of methanolic, ethanolic, and aqueous extracts of *Salvia rosmarinus* harvested in Lastva Grbaljska, Montenegro, across three seasons (L3N, L3M, and L3J) and tested at various concentrations (1, 10, 50, 100, 250, and 500 μg/mL, x-axis), for 24 and 72 h, assessed using the MTT assay on the colorectal cancer cell line, HCT-116; Figure S3: Radar plots generated from IBR analysis illustrating the overall bioactivity of methanolic, ethanolic, and aqueous extracts of *Salvia rosmarinus* harvested from Lastva Grbaljska, Montenegro (L3), across three seasons (N—November; M—March; J—July). The plots are based on data obtained from four antioxidant assays: DPPH, FRAP, total reducing power (TRP), and β-carotene bleaching, three enzyme-inhibitory assays: α-glucosidase, acetylcholinesterase (AChE), and tyrosinase (TYR) inhibition, as well as a cytotoxic assay, MTT, on colorectal cancer cell line, HCT-116. The area enclosed by each radar plot represents the overall tested bioactivity of the sample: the larger the area, the higher the overall biological activity; Table S1: GC-MS profile of volatile terpenes in *Salvia rosmarinus* extracts; Table S2: Peak areas of compounds identified in *Salvia rosmarinus* extracts; values were obtained from full scan MS. Table S3: LC/MS data on specialized metabolites identified in rosemary samples. Table S4: The origin and harvesting dates of the plant material used in this study, and the respective voucher numbers.

## Abbreviations

The following abbreviations are used in this manuscript:

ROS	Reactive oxygen species
5-FU	5-Fluorouracil
L1N	Rosemary harvested in Belgrade, in November
L1M	Rosemary harvested in Belgrade, in March
L1J	Rosemary harvested in Belgrade, in July
L2N	Rosemary harvested in Bogatić, in November
L2M	Rosemary harvested in Bogatić, in March
L2J	Rosemary harvested in Bogatić, in July
L3N	Rosemary harvested in Lastva Grbaljska, in November
L3M	Rosemary harvested in Lastva Grbaljska, in March
L3J	Rosemary harvested in Lastva Grbaljska, in July
DPPH	2,2-Diphenyl-1-picrylhydrazyl
FRAP	Ferric reducing ability of plasma
TRP	Total reducing power
AChE	Acetylcholinesterase
TYR	Tyrosinase
TPC	Total phenolic content
TFC	Total flavonoid content
FC	Flavonol content
PAC	Phenolic acid content
