# Supplementary material for: Therapeutic Potential of Salvia rosmarinus: Seasonal and Geographical Variation in Phytochemical Composition, Bioactivity, and Synergistic Effects of Rosmarinic Acid with 5-FU
Source: Plants (Basel). 2025 Dec 19;15(1):1. doi: 10.3390/plants15010001 (PMC12787868; doi:10.3390/plants15010001)
Supplement: Supplementary file 1 [file plants-15-00001-s001.zip › Table S3.pdf]

**Table S3**. LC/MS data on specialized metabolites identified in rosemary samples.

| No | Compound name | $t_R$, min | Molecular formula, [M–H]$^-$ | Calculated mass, M–H]$^-$ | Exact mass, M–H]– | Δ ppm | MS$^2$ Fragments, (% Base Peak) | ref. *Rosmarinus* |
|---|---|---|---|---|---|---|---|---|
| *Hydroxybenzoic acid derivatives* | | | | | | | | |
| **1** | **Galloyl hexoside** | 0.55 | C13H15O10– | 331.06707 | 331.06641 | 1.98 | 125.02415(51), 149.99565(4), 168.00606(86), 169.01384(31), 313.05597(21), **331.06647**(100) | NA |
| **2** | **Vanilloyl hexoside** | 0.73 | C14H17O9– | 329.08781 | 329.08695 | 2.59 | 108.02144(11), 123.04485(29), 152.01109(16), **167.03455**(100) | [18] |
| **3** | **Dihydroxybenzoyl hexoside** | 0.79 | C13H15O9– | 315.07216 | 315.07126 | 2.83 | 152.01122(9), **153.02029**(100), 315.07132(7) | [19] |
| **4** | **Hydroxybenzoyl hexoside** | 0.98 | C13H15O8– | 299.07724 | 299.07641 | 2.77 | 93.03437(29), **137.02412**(100) | [20] |
| **5** | **Dihydroxybenzoic acid** | 1.11 | C7H5O4– | 153.01933 | 153.01896 | 2.45 | **109.02927**(100), 153.01900(35) | [21] |
| **6** | **Hydroxybenzoic acid isomer 1** | 2.24 | C7H5O3– | 137.02442 | 137.02410 | 2.28 | 93.03439(4), **137.02415**(100) | [21] |
| **7** | **Hydroxybenzoic acid isomer 2** | 6.69 | C7H5O3– | 137.02442 | 137.02405 | 2.70 | **93.03436**(100), 137.02412(74) | [21] |
| **8** | **Galloyl-hydroxybenzoyl hexoside** | 7.15 | C20H19O12– | 451.08820 | 451.08665 | 3.43 | 137.02414(80), 168.00607(75), 169.01370(13), 313.05585(28), **331.06641**(100) | NA |
| *Hydroxycinnamic acid derivatives* | | | | | | | | |
| **9** | **Caffeic acid** | 4.87 | C9H7O4– | 179.03498 | 179.03453 | 2.52 | **135.04488**(100), 179.03467(24) | [21] |
| **10** | **Aesculetin** | 5.04 | C9H5O4– | 177.01933 | 177.01888 | 2.54 | 105.03440(3), 133.02916(11), **177.01884**(100) | NA |
| **11** | ***p*-Coumaroyl hexoside** | 5.28 | C15H17O8– | 325.09289 | 325.09208 | 2.51 | 119.04996(50), 145.02917(9), **163.03967**(100) | [18] |
| **12** | ***p*-Coumaric acid** | 5.28 | C9H7O3– | 163.04007 | 163.03966 | 2.48 | **119.05006**(100), 163.03989(12) | [21] |
| **13** | **4-Caffeoylquinic acid** | 5.36 | C16H17O9– | 353.08781 | 353.08676 | 2.97 | 135.04494(32), **173.04521**(100), 179.03467(77), 191.05571(53) | [22] |
| **14** | **4-*p*-Coumaroylquinic acid** | 5.79 | C16H17O8– | 337.09289 | 337.09189 | 2.97 | 119.04992(7), 163.03970(22), **173.04510**(100), 191.05544(3) | [19] |
| **15** | **Caffeoylshikimic acid** | 5.85 | C16H15O8– | 335.07724 | 335.07653 | 2.11 | 135.04494(47), 161.02425(23), **179.03465**(100) | NA |
| **16** | **Feruloyl hexoside** | 5.89 | C16H19O9– | 355.10346 | 355.10205 | 3.97 | 134.03709(21), 149.06056(25), 175.03972(62), **193.05028**(100), 235.06075(65), 295.08191(22) | [23] |
| **17** | **Sinapoyl hexoside** | 5.92 | C17H21O10– | 385.11402 | 385.11267 | 3.50 | 164.04761(33), 179.07104(27), 208.03741(13), **223.06071**(100), 265.07117(65), 325.09195(20) | [20] |
| **18** | **Prolithospermic acid** | 5.95 | C18H13O8– | 357.06159 | 357.06016 | 4.00 | **109.02929**(100), 135.04478(6), 147.04489(7), 159.04485(46), 203.03456(27), 269.08142(64) | [24] |
| **19** | **4-Feruloylquinic acid** | 6.05 | C17H19O9– | 367.10346 | 367.10243 | 2.78 | 134.03690(8), **173.04512**(100), 193.05013(23) | NA |

| | | | | | | | | |
|---|---|---|---|---|---|---|---|---|
| **20** | **Lithospermic acid** | 6.26 | $C_{27}H_{21}O_{12}^{-}$ | 537.10385 | 537.10228 | 2.92 | **109.02925**(100), 135.04488(41), 179.03458(72), 185.02400(89), 197.04521(23), 295.06049(89) | [25] |
| **21** | **Salviaflaside** | 6.28 | $C_{24}H_{25}O_{13}^{-}$ | 521.13007 | 521.12865 | 2.71 | 135.04491(20), **161.02405**(100), 179.03461(47), 197.04504(89), 323.07654(50), 359.09808(21) | [22] |
| **22** | **Ferulic acid** | 6.32 | $C_{10}H_{9}O_{4}^{-}$ | 193.05063 | 193.05008 | 2.85 | **134.03706**(100), 149.06042(17), 178.02679(58), 193.05003(16) | [21] |
| **23** | **Yunnaneic acid D** | 6.34 | $C_{27}H_{23}O_{12}^{-}$ | 539.11950 | 539.11802 | 2.74 | 135.04485(79), **161.02402**(100), 179.03456(52), 197.04506(57), 279.06534(13), 297.07605(49) | [25] |
| **24** | **Yunnaneic acid F** | 6.34 | $C_{29}H_{25}O_{14}^{-}$ | 597.12498 | 597.12315 | 3.05 | **135.04475**(100), 179.03450(19), 197.04494(28), 267.10181(36), 293.08109(7), 311.09161(17) | [25] |
| **25** | **Salvianolic acid B** | 6.58 | $C_{36}H_{29}O_{16}^{-}$ | 717.14611 | 717.14424 | 2.61 | **161.02400**(100), 179.03453(12), 197.04503(39), 295.06058(11), 339.04971(18), 359.07556(3) | [21] |
| **26** | **Rosmarinic acid** | 6.61 | $C_{18}H_{15}O_{8}^{-}$ | 359.07724 | 359.07659 | 1.81 | 135.04480(7), **161.02403**(100), 179.03464(19), 197.04500(35) | [21] |
| **27** | **Sagerinic acid** | 6.70 | $C_{36}H_{31}O_{16}^{-}$ | 719.16176 | 719.15977 | 2.76 | 135.04486(3), **161.02402**(100), 179.03447(10), 197.04503(41) | [22] |
| **28** | **Salvianolic acid** | 6.87 | $C_{26}H_{21}O_{10}^{-}$ | 493.11402 | 493.11256 | 2.97 | 109.02927(89), 135.04491(42), **185.02403**(100), 197.04510(23), 295.06061(83), 313.07117(7) | [22] |
| **29** | **Methyl caffeate** | 6.89 | $C_{10}H_{9}O_{4}^{-}$ | 193.05063 | 193.05009 | 2.82 | 134.03702(28), 161.02405(16), 178.02654(5), **193.05009**(100) | NA |
| **30** | **Di-dihydrocaffeoylquinic acid** | 6.98 | $C_{25}H_{27}O_{12}^{-}$ | 519.15080 | 519.14920 | 3.08 | 137.06044(14), **181.05009**(100) | NA |
| **31** | **Caffeoyl-dihydrocaffeoylquinic acid** | 7.05 | $C_{25}H_{25}O_{12}^{-}$ | 517.13515 | 517.13354 | 3.12 | 135.04486(20), **161.02406**(100), 175.03966(12), 179.03464(50) | NA |
| **32** | **Methyl rosmarinate** | 7.14 | $C_{19}H_{17}O_{8}^{-}$ | 373.09289 | 373.09172 | 3.14 | **135.04478**(100), 160.01604(12), 175.03958(78), 179.03450(32), 193.04962(5), 197.04500(56) | [21] |
| **33** | **Methyl lithospermate** | 7.32 | $C_{28}H_{23}O_{12}^{-}$ | 551.11950 | 551.11779 | 3.11 | 135.02974(17), **151.03961**(100), 161.02397(37), 267.10117(54), 293.08099(35), 311.09183(73) | NA |
| **34** | **Ethyl caffeate** | 7.49 | $C_{11}H_{11}O_{4}^{-}$ | 207.06628 | 207.06575 | 2.59 | 134.03700(19), 135.04480(17), 161.02400(15), 179.03458(27), **207.06572**(100) | NA |
| **35** | **Orthosiphoic acid A** | 7.65 | $C_{27}H_{21}O_{11}^{-}$ | 521.10894 | 521.10780 | 2.17 | 135.04497(5), **161.02397**(100), 163.03944(5), 179.03477(10), 197.04495(32) | NA |
| **36** | **Nepetoidin B** | 7.83 | $C_{17}H_{13}O_{6}^{-}$ | 313.07176 | 313.07112 | 2.07 | 133.02928(2), 151.03976(8), **161.02408**(100) | [26] |
| **37** | **Salvianolic acid C** | 7.87 | $C_{26}H_{19}O_{10}^{-}$ | 491.09837 | 491.09709 | 2.61 | 135.04488(10), 149.02422(4), 161.02406(18), **179.03455**(100), 267.06573(13) | [25] |
| **38** | **Methyl-salvianolic acid C** | 8.63 | $C_{27}H_{21}O_{10}^{-}$ | 505.11402 | 505.11271 | 2.60 | 149.02451(5), 161.02400(28), 179.03413(4), **193.05019**(100), 267.06552(22), 311.05560(10) | NA |
| | | | | *Flavonoid glycosides* | | | | |

| | | | | | | | | |
|---|---|---|---|---|---|---|---|---|
| **39** | **Naringenin 6,8-di-C-hexoside** | 5.57 | C27H31O15– | 595.16684 | 595.16504 | 3.03 | 313.07065(14), 325.07034(4), 355.08087(95), **385.09128**(100), 397.09149(11), 415.10233(31) | NA |
| **40** | **Quercetin 3-O-(6"-rhamnosyl)-hexoside (Rutin)** | 6.06 | C27H29O16– | 609.14611 | 609.14456 | 2.54 | 300.02686(10), **301.03430**(100), 609.14447(12) | [27] |
| **41** | **Quercetin 3-O-hexoside (Hyperoside)** | 6.10 | C21H19O12– | 463.08820 | 463.08691 | 2.79 | 300.02698(49), **301.03464**(100), 463.08691(14) | [28] |
| **42** | **Luteolin 3'-O-hexoside-7-O-hexuronide** | 6.23 | C27H27O17– | 623.12537 | 623.12457 | 1.29 | 113.02394(3), 161.02383(5), 284.03210(4), **285.03952**(100), 447.09161(88), 461.07040(4) | NA |
| **43** | **Luteolin 7-O-(6"-rhamnosyl)-hexoside** | 6.30 | C27H29O15– | 593.15119 | 593.14987 | 2.23 | **285.03989**(100), 593.15033(18) | [22] |
| **44** | **Nepetin 7-O-(6"-rhamnosyl)-hexoside** | 6.39 | C28H31O16– | 623.16176 | 623.16042 | 2.14 | 299.01901(10), 300.02682(19), 314.04227(4), **315.05026**(100) | [29] |
| **45** | **Kaempferol 3-O-hexoside (Astragalin)** | 6.37 | C21H19O11– | 447.09329 | 447.09174 | 3.45 | **284.03183**(100), 285.03949(85), 447.09161(52) | [19] |
| **46** | **Nepetin 7-O-hexoside (Nepitrin)** | 6.42 | C22H21O12– | 477.10385 | 477.10230 | 3.25 | 300.02692(40), 301.03500(10), 314.04297(19), **315.04645**(100), 462.08023(7) | [30] |
| **47** | **Quercetin 3-O-(6"-caffeoyl)-hexoside** | 6.49 | C30H25O15– | 625.11989 | 625.11844 | 2.33 | 135.04482(2), 161.02408(4), 179.03471(2), 300.02682(4), **301.03458**(100) | NA |
| **48** | **Eriodictyol 7-O-(6"-rhamnosyl)-hexoside (Eriocitrin)** | 6.49 | C27H31O15– | 595.16684 | 595.16630 | 0.92 | 135.04486(23), **151.00336**(100), 287.05542(55) | [19] |
| **49** | **Nepetin 7-O-hexuronide** | 6.51 | C22H19O13– | 491.08311 | 491.08199 | 2.29 | 161.02428(12), 300.02698(77), **315.04996**(100) | NA |
| **50** | **Diosmetin 7-O-(6"-rhamnosyl)-hexoside (Diosmin)** | 6.62 | C28H31O15– | 607.16684 | 607.16549 | 2.22 | 284.03204(28), **299.05545**(100) | [28] |
| **51** | **Hesperetin 7-O-(6"-rhamnosyl)-hexoside (Hesperidin)** | 6.63 | C28H33O15– | 609.18249 | 609.18113 | 2.24 | 285.03976(12), 286.04755(6), **301.07101**(100) | [28] |
| **52** | **Apigenin 7-O-hexoside** | 6.66 | C21H19O10– | 431.09837 | 431.09664 | 4.02 | 135.04486(12), 268.03711(99), **269.04486**(100), 431.09708(89) | [22] |
| **53** | **Apigenin 7-O-hexuronide** | 6.70 | C21H17O11– | 445.07764 | 445.07589 | 3.91 | **269.04492**(100) | [20] |
| **54** | **Chrysoeriol 7-O-hexuronide** | 6.76 | C22H19O12– | 475.08820 | 475.08695 | 2.62 | 113.02413(48), 161.02431(13), 175.03981(17), **284.03195**(100), 299.05530(92) | [31] |
| **55** | **Nepetin 7-O-(6"-caffeoyl)-hexoside** | 6.80 | C31H27O15– | 639.13554 | 639.13383 | 2.68 | 161.02396(12), 179.03441(6), 300.02667(38), 301.03394(6), **315.05011**(100) | NA |
| **56** | **Luteolin 3'-O-hexuronide** | 6.81 | C21H17O12– | 461.07255 | 461.07114 | 3.05 | **285.03940**(100) | [32] |

| | | | | | | | | |
|---|---|---|---|---|---|---|---|---|
| **57** | **Cirsiliol 4′-O-hexoside** | 6.91 | C23H23O12– | 491.11950 | 491.11835 | 2.34 | 314.04233(33), **329.06592**(100) | NA |
| **58** | **Luteolin 7-O-(6"-feruloyl)-hexoside** | 6.97 | C31H27O14– | 623.14063 | 623.13913 | 2.40 | 161.02394(6), 284.03195(8), **285.03964**(100) | NA |
| **59** | **Luteolin 7-O-(6"-*p*-coumaroyl)-hexoside** | 7.00 | C30H25O13– | 593.13007 | 593.12853 | 2.59 | **285.03958**(100) | NA |
| **60** | **Luteolin 3'-O-(2"-acetyl)-hexuronide** | 7.08 | C23H19O13– | 503.08311 | 503.08162 | 2.97 | 284.03198(5), **285.03955**(100) | [32] |
| **61** | **Nepetin 7-O-(6"-*p*-coumaroyl)-hexoside** | 7.14 | C31H27O14– | 623.14063 | 623.13899 | 2.64 | 299.01917(26), 300.02689(36), 314.04239(6), **315.05038**(100), 623.13922(25) | [23] |
| **62** | **Chrysoeriol 7-O-(6′′-*p*-coumaroyl)-hexoside** | 7.36 | C31H27O13– | 607.14572 | 607.14464 | 1.77 | 145.02916(10), 269.04501(11), 283.02426(3), 284.03201(40), **299.05536**(100) | NA |
| **63** | **Apigenin 7-O-(6′′-*p*-coumaroyl)-hexoside** | 7.45 | C30H25O12– | 577.13515 | 577.13392 | 2.13 | 101.0242(3), 145.02916(9), 161.02405(3), 163.03957(3), **269.04504**(100) | NA |
| **64** | **Hispidulin 7-O-hexoside (Homoplantaginin)** | 7.52 | C22H21O11– | 461.10894 | 461.10728 | 3.58 | 284.03165(5), 285.03967(43), **299.05539**(100) | [22] |
| **65** | **Dihydroxy-trimethoxyflavone O-(malonyl)-hexoside** | 7.54 | C27H27O15– | 591.13554 | 591.13464 | 1.53 | 137.02403(31), 163.03984(49), 179.03497(22), 313.03540(10), **328.05801**(100), 343.08160(20) | NA |
| **66** | **Cirsimaritin 7-O-(6"-acetyl)-hexoside** | 7.63 | C25H25O12– | 517.13515 | 517.13385 | 2.51 | 283.02454(8), 285.03986(7), **298.04776**(100), 313.07129(79) | NA |
| **67** | **Acacetin 7-O-hexoside** | 7.65 | C22H21O10– | 445.11402 | 445.11268 | 3.01 | 135.02953(10), 161.02415(7), 268.03711(4), **283.06046**(100) | NA |
| **68** | **Luteolin 7-O-(6′′-cinnamoyl)-hexoside** | 7.79 | C30H25O12– | 577.13515 | 577.13378 | 2.38 | **284.03201**(100), 285.04001(22) | NA |
| **69** | **Cirsimaritin 7-O-(6"-hydroxybenzoyl)-hexoside** | 7.84 | C30H27O13– | 595.14572 | 595.14451 | 2.02 | 135.04509(4), 161.02394(10), 179.03439(4), 283.02426(19), 298.04733(33), **313.07101**(100) | NA |
| | | | | *Flavonoid aglycones* | | | | |
| **70** | **Luteolin** | 7.29 | C15H9O6– | 285.04046 | 285.03973 | 2.55 | **285.03983**(100) | [33] |
| **71** | **Nepetin** | 7.32 | C16H11O7– | 315.05103 | 315.05034 | 2.19 | **300.02667**(100), 301.03052(2), 315.05032(11) | [34] |
| **72** | **Apigenin** | 7.69 | C15H9O5– | 269.04555 | 269.04498 | 2.12 | 149.02402(2), 151.00307(3), 225.05467(2), **269.04471**(100) | [22] |
| **73** | **Diosmetin** | 7.72 | C16H11O6– | 299.05611 | 299.05551 | 2.01 | **284.03198**(100), 299.05566(13) | [22] |
| **74** | **Hesperetin** | 7.76 | C16H13O6– | 301.07176 | 301.07104 | 2.39 | 151.00327(18), 164.01112(37), 242.05786(25), 257.08118(16), 286.04739(44), **301.07104**(100) | [27] |
| **75** | **Daidzein** | 8.18 | C15H9O4– | 253.05063 | 253.05003 | 2.36 | 121.02917(6), 181.06656(4), **253.05031**(100) | [27] |
| **76** | **Cirsimaritin** | 8.25 | C17H13O6– | 313.07176 | 313.07099 | 2.46 | 161.02399(5), 269.04468(5), **283.02405**(100), 297.03967(15), 298.04745(100), 313.07101(36) | [35] |

| | | | | | | | | |
|---|---|---|---|---|---|---|---|---|
| **77** | **Xanthomicrol** | 8.33 | C18H15O7– | 343.08233 | 343.08113 | 3.48 | 313.03458(51), **328.05798**(100) | [19] |
| **78** | **Genkwanin** | 8.71 | C16H11O5– | 283.06120 | 283.06051 | 2.42 | 268.03696(73), **283.06033**(100) | [36] |
| | | | | *Terpenoids* | | | | |
| **79** | **Asiatic acid** | 9.13 | C30H47O5– | 487.34290 | 487.34144 | 3.00 | 467.31384(3), 469.33102(28), **487.34140**(100) | [22] |
| **80** | **Carnosol** | 9.72 | C20H25O4– | 329.17583 | 329.17521 | 1.90 | **285.18539**(100), 286.18893(3) | [21] |
| **81** | **Augustic acid** | 10.13 | C30H47O4– | 471.34798 | 471.34654 | 3.06 | **471.34644**(100) | [21] |
| **82** | **Carnosic acid** | 10.36 | C20H27O4– | 331.19148 | 331.19037 | 3.36 | **287.20078**(100), 331.1907(20) | [21] |
| **83** | **Ursolic acid** | 11.32 | C30H47O3– | 455.35307 | 455.35149 | 3.46 | **455.35120**(100) | [21] |
| | | | | *Other metabolites* | | | | |
| **84** | **Dehydroascorbic acid** | 0.60 | C6H5O6– | 173.00916 | 173.00874 | 2.44 | **85.02929**(100), 111.00854(77), 129.01907(17) | [37] |
| **85** | **Salvianic acid A** | 0.84 | C9H9O5– | 197.04555 | 197.04495 | 3.03 | **72.99294**(100), 123.04486(55), 135.04482(75), 179.03458(49), 197.04507(8) | [35] |
| **86** | **Hydroxytyrosol hexoside** | 0.88 | C14H19O8– | 315.10854 | 315.10774 | 2.53 | 123.04491(15), **153.05534**(100) | NA |
| **87** | **Sinapyl alcohol** | 5.37 | C11H13O4– | 209.08193 | 209.08140 | 2.53 | 161.02408(48), 176.04755(35), 179.03477(72), 191.07114(8), **194.05817**(100), 209.08177(9) | [38] |
| **88** | **Eugenol** | 7.31 | C10H11O2– | 163.07645 | 163.07606 | 2.41 | **148.05270**(100), 163.07610(6) | [39] |
| **89** | **Rosmanol** | 8.28 | C20H25O5– | 345.17075 | 345.16930 | 4.20 | 283.16940(79), 301.18036(6), **345.16910**(100) | [28] |
| **90** | **Resveratrol** | 9.03 | C14H11O3– | 227.07137 | 227.07074 | 2.75 | **183.08113**(100), 227.07094(29) | [40] |
| **91** | **Rosmadial** | 10.00 | C20H23O5– | 343.15510 | 343.15360 | 4.36 | **299.16449**(100), 300.16925(4), 343.15384(59) | [22] |
| **92** | **Rosmaridiphenol** | 10.61 | C20H27O3– | 315.19657 | 315.19544 | 3.57 | 285.18527(43), 287.19415(2), **315.19547**(100) | [21] |

NA – not assessed previously.
