# Supplementary material for: Therapeutic Potential of Salvia rosmarinus: Seasonal and Geographical Variation in Phytochemical Composition, Bioactivity, and Synergistic Effects of Rosmarinic Acid with 5-FU
Source: Plants (Basel). 2025 Dec 19;15(1):1. doi: 10.3390/plants15010001 (PMC12787868; doi:10.3390/plants15010001)
Supplement: Supplementary file 1 [file plants-15-00001-s001.zip › Table S4.pdf]

Table S4. The origin and harvesting dates of the plant material used in this study and the respective voucher numbers.

Plant species	Locality (L)	Coordinates	Altitude	Harvesting date	Voucer number
Rosmarinus officinalis L.	Belgrade	44.8517° N, 20.4697° E	72 m	November 15th, 2016	17 343
				March 15th, 2017	17 344
				July 15th, 2017	17 345
	Bogatić	44.8375° N, 19.4806° E	81 m	November 15th, 2016	17 346
				March 15th, 2017	17 347
				July 15th, 2017	17 348
	Lastva Grbaljska	42.3105° N, 18.8032° E	22 m	November 15th, 2016	17 349
				March 15th, 2017	17 350
				July 15th, 2017	17 351
