# Supplementary figures and images for: Therapeutic Potential of Salvia rosmarinus: Seasonal and Geographical Variation in Phytochemical Composition, Bioactivity, and Synergistic Effects of Rosmarinic Acid with 5-FU
Source: Plants (Basel). 2025 Dec 19;15(1):1. doi: 10.3390/plants15010001 (PMC12787868; doi:10.3390/plants15010001)

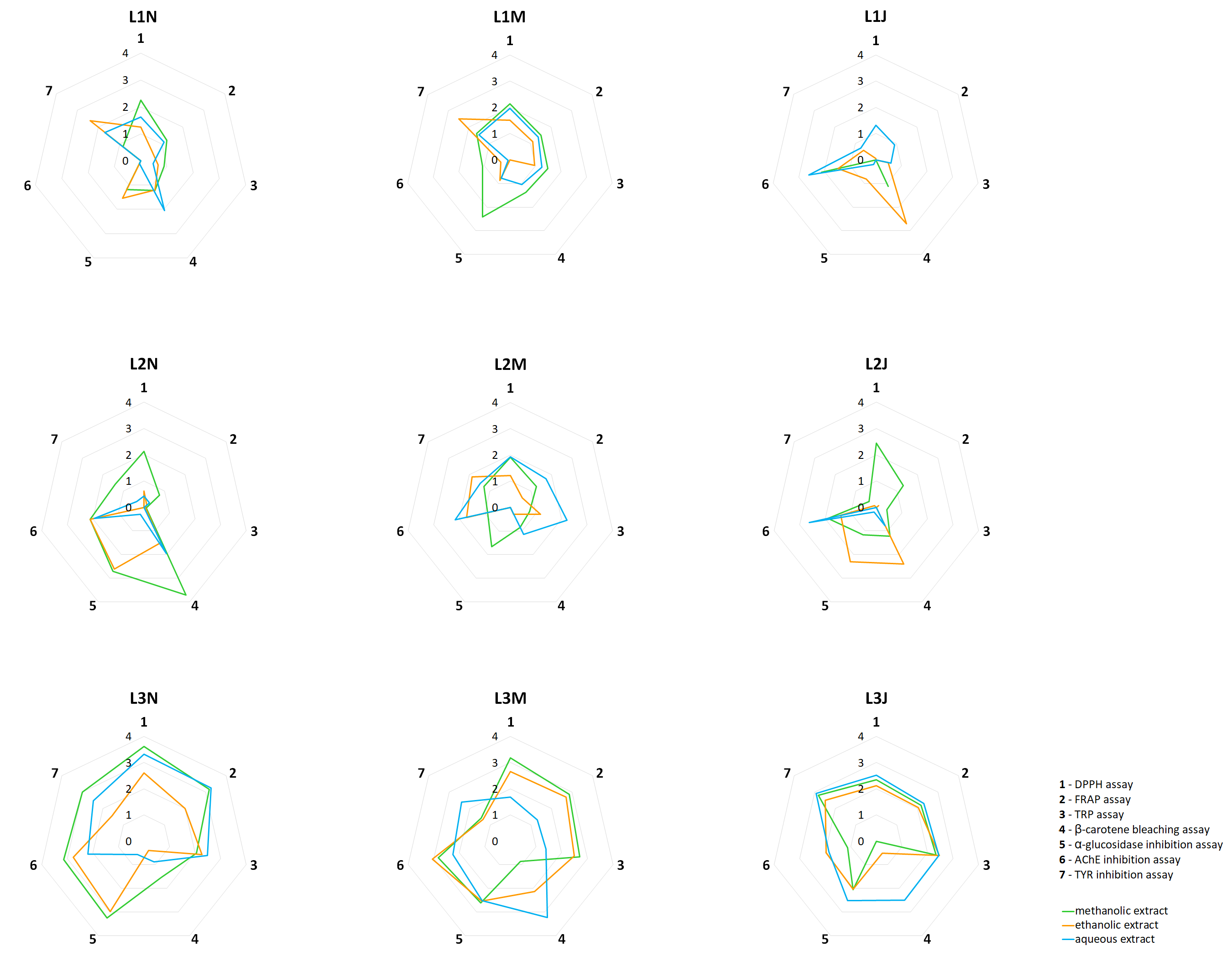

Supplement: Supplementary file 1 [file plants-15-00001-s001.zip › Figure S1.tif]

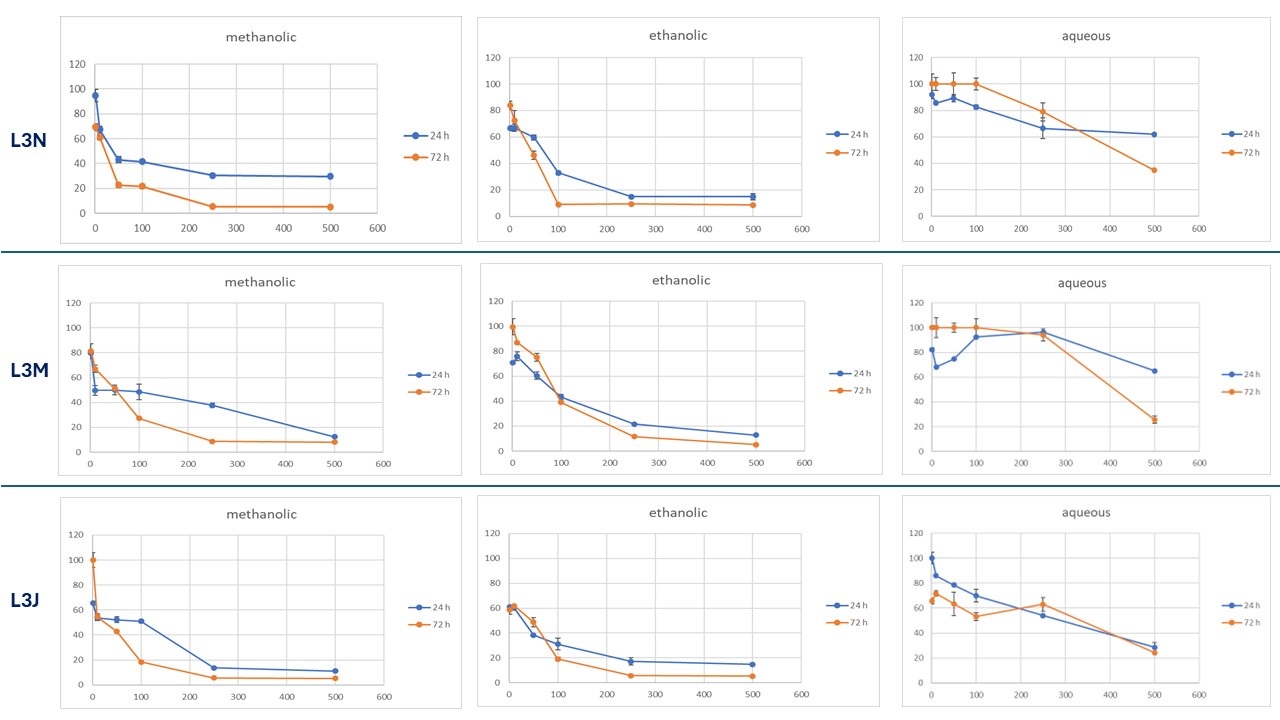

Supplement: Supplementary file 1 [file plants-15-00001-s001.zip › Figure S2.jpg]

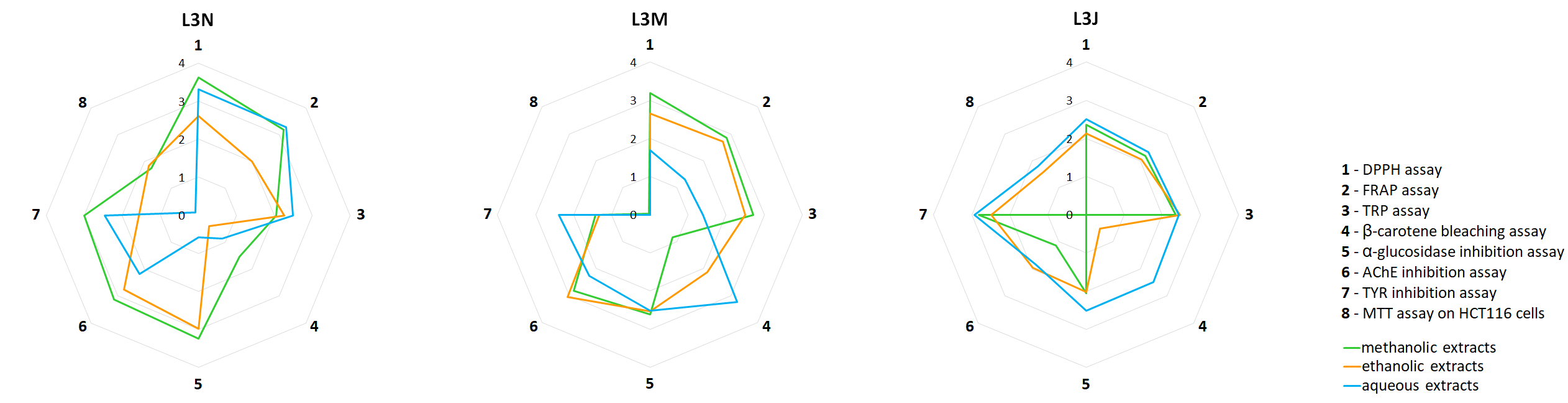

Supplement: Supplementary file 1 [file plants-15-00001-s001.zip › Figure S3.tif]
